# Identification of *Pueraria* spp. through DNA barcoding and comparative transcriptomics

**DOI:** 10.1186/s12870-021-03383-x

**Published:** 2022-01-03

**Authors:** Laci M. Adolfo, Xiaolan Rao, Richard A. Dixon

**Affiliations:** 1 BioDiscovery Institute and Department of Biological Sciences, University of North Texas, 1155 Union Circle #305220, Denton, TX 76203-5017 USA; 2 College of Life Sciences, Hubei University, Wuhan, 430068 Hubei Province China

**Keywords:** Kudzu, DNA barcoding, Microsatellites, Comparative transcriptomics

## Abstract

**Background:**

Kudzu is a term used generically to describe members of the genus *Pueraria*. Kudzu roots have been used for centuries in traditional Chinese medicine in view of their high levels of beneficial isoflavones including the unique 8-*C-*glycoside of daidzein, puerarin. In the US, kudzu is seen as a noxious weed causing ecological and economic damage. However, not all kudzu species make puerarin or are equally invasive. Kudzu remains difficult to identify due to its diverse morphology and inconsistent nomenclature.

**Results:**

We have generated sequences for the internal transcribed spacer 2 (ITS2) and maturase K (matK) regions of *Pueraria montana lobata*, *P. montana montana*, and *P. phaseoloides*, and identified two accessions previously used for differential analysis of puerarin biosynthesis as *P. lobata* and *P. phaseoloides*. Additionally, we have generated root transcriptomes for the puerarin-producing *P. m. lobata* and the non-puerarin producing *P. phaseoloides.* Within the transcriptomes, microsatellites were identified to aid in species identification as well as population diversity.

**Conclusions:**

The barcode sequences generated will aid in fast and efficient identification of the three kudzu species. Additionally, the microsatellites identified from the transcriptomes will aid in genetic analysis. The root transcriptomes also provide a molecular toolkit for comparative gene expression analysis towards elucidation of the biosynthesis of kudzu phytochemicals.

**Supplementary Information:**

The online version contains supplementary material available at 10.1186/s12870-021-03383-x.

## Summary

Various kudzu accessions were analyzed through barcoding and comparative transcriptomics, generating tools for identification and molecular pathway analysis.

## Background

Kudzu has been used in traditional Chinese medicine with the roots being considered the most valuable part of the plant [1]. The high levels of isoflavones in the roots are believed to be important for the medicinal properties of kudzu [2]. Kudzu contains the same major isoflavones that are found in other legumes, including the aglycones daidzein, genistein, and formononetin as well as their *O-*glycosides daidzin, genistin, and ononin. However, kudzu also contains puerarin, the 8-*C*-glycoside of daidzein [3]. Many of the health benefits of kudzu are believed to come from puerarin, because the carbon-carbon glycosidic bond in puerarin makes it resistant to hydrolysis when ingested [2]. However, health benefits have also been linked to daidzin and genistin, as well as the methylated isoflavone formononetin and its glycoside, ononin. A Chinese pharmacopeia dating back to 200 B.C. mentions the roots of kudzu and their use in various treatments. Kudzu was administered to help with a range of ailments including inflammation, diarrhea, and even alcoholism [4]. In its native habitat, Asia, kudzu grows well with growth being controlled by pests and climate. In the US, kudzu is an invasive weed, especially in the southeast [5].

Mass planting of kudzu allowed it to spread rapidly throughout the Southeast US, where the climate is perfect for it, with high temperatures and plenty of rainfall, and natural predators are absent. Kudzu vines can grow up to 12 in. a day. Kudzu out-competed native flora and caused an economic burden as the vines crept up utility poles and disrupted power [5]. The removal of kudzu is a difficult process as simply removing the top foliage does not stop the spread of the plant; kudzu’s extensive root system includes a large tap root from which many roots and vines sprout [6, 7]). The US federal government declared kudzu a federal noxious weed in the mid to late 1990’s. It was eventually removed from the federal noxious weed list; however, it is still on the noxious weed lists of several states, including Texas [7].

The taxonomy of kudzu is unclear, with multiple synonyms and multiple varieties within species, such as *Pueraria montana*, *P. thomsonii*, and *P. lobata* which can also be referred to as *P. montana var. montana*, *P. montana var. chinensis*, and *P. montana var. lobata*, respectively. The classification as different species and different variants has been confusing, especially as the morphological characteristics of these individual varieties are highly variable [8, 9].

The availability of established DNA barcodes that can differentiate between different species/varieties would allow for positive identification of kudzu in the wild, and could aid ecological studies; for example, fecal samples are often examined to determine the dietary behavior of animals and insects [10–12]. Furthermore, DNA barcoding could facilitate quality control and assurance for herbal supplements [13–15].

A previous study used kudzu accessions collected in the field (Ardmore, OK) and obtained commercially (Kudzu Kingdom, Kodak, TN) to interrogate puerarin biosynthesis through differential expression analysis following EST sequencing [16]. To aid the identification of these and other kudzu accessions, we have generated barcodes for the ITS2 and matK regions of three kudzu species/varieties. We have also generated transcriptomic data of the roots of the puerarin producing *P. m. lobata* and the non-puerarin producing *P. phaseoloides*. The transcriptomic data generated allows for differential gene expression analysis and also identifies simple sequence repeat (SSRs) markers between the two kudzu species. These genomic resources will serve as references for identifying kudzu species for eradication, harvesting of phytochemicals, validation of supplements, and ecological research. Additionally, the comparative transcriptomics provides a molecular resource for exploring genes active in the synthesis of valuable phytochemicals.

## Results

### Seed morphology

**Fig. 1:**
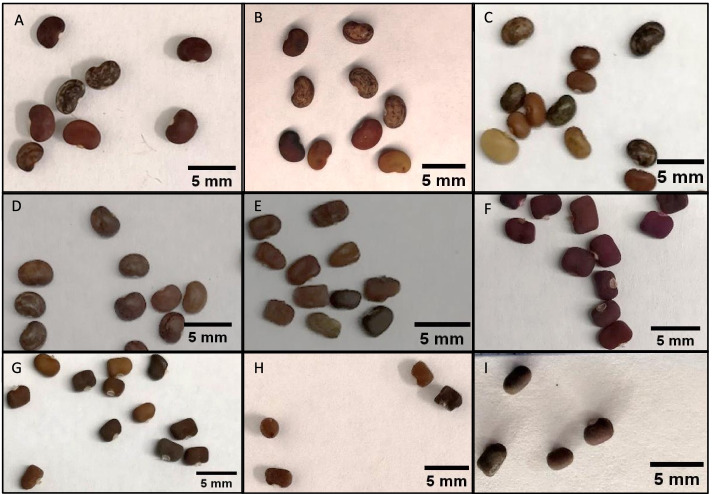
Morphology of seeds from each kudzu accession. **A** Oklahoma (wild); **B** Texas (wild); **C** PI 9227 (*P. m. lobata*); **D** PI 434246 (*P. m. lobata*); **E** PI 298615 (*P. m. montana*); **F** Kudzu Kingdom (commercial); **G** BRSEEDS (commercial); **H** PI 308576 (*P. phaseoloides*); **I** DLEG 890244 (*P. phaseoloides*)

The origins of the kudzu accessions analyzed in the present work are provided in the Methods. Wild kudzu collected from Oklahoma and Texas, and USDA PI 434246 and PI 9227 all had kidney-shaped seeds. Most of the seeds were dark brown with a few being lighter brown to reddish. The seeds also had lighter colored striations. They measured approximately 3.2 mm in length (Fig. 1A-D). The Kudzu Kingdom, BRSEEDS, USDA PI 308576, and USDA DLEG 890244 seeds were rectangular to oblong. The seed colors ranged from maroon to orange to golden yellow and were also approximately 3.2 mm in length (Fig. 1F-I). The USDA PI 298615 seeds were rectangular to oblong, and dark to medium brown in color. They were smaller than the other seeds, measuring approximately 2.1 mm in length (Fig. 1E).

### Plant morphology

**Fig. 2:**
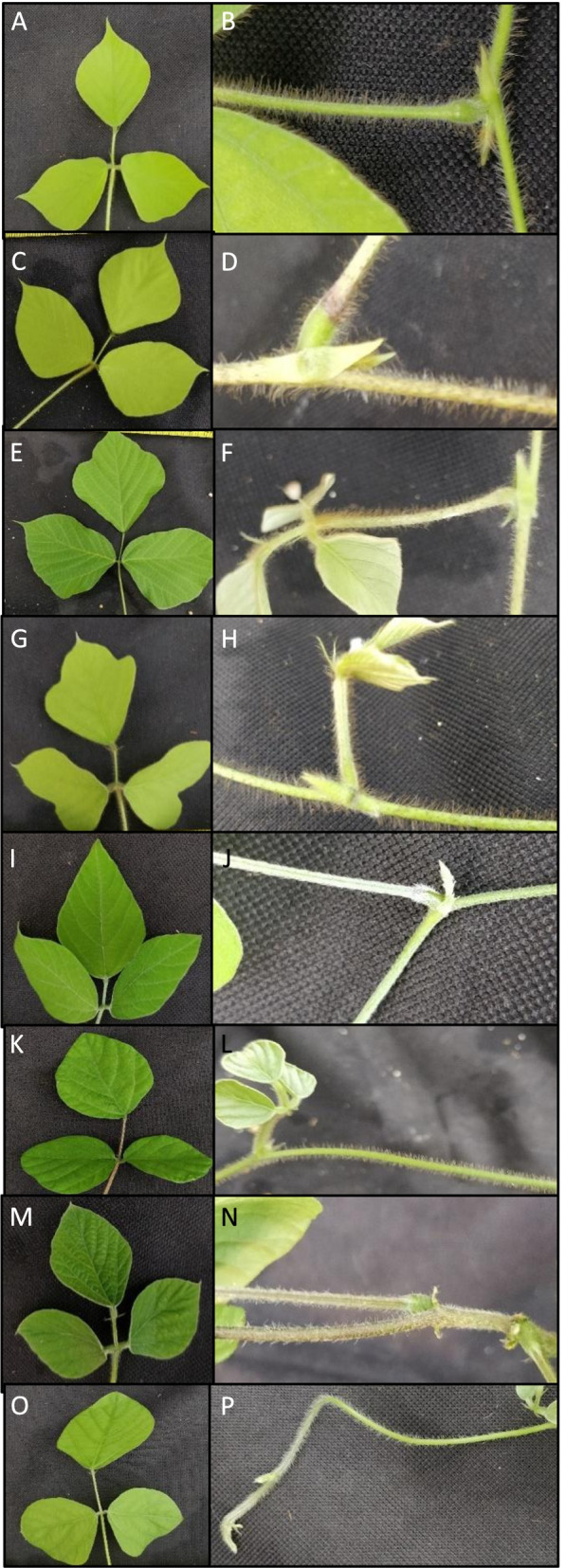
Images of vines, leaves, and trichomes for each plant accession. **A**-**B** Oklahoma (wild); **C**-**D**, Texas (wild); **E**-**F** PI 9227 (*P. m. lobata*); **G**-**H** PI 434246 (*P. m. lobata*); **I**-**J**, PI 298615 (*P. m. montana*); **K**-**L** Kudzu Kingdom (commercial); **M**-**N** BRSEEDS (commercial); **O**-**P** PI 308576 (*P. phaseoloides*)

All plants grew as vines with trifoliate leaves and trichomes present on the leaves and stems/vines (Supplemental Fig. 1). DLEG 890244 (*P. phaseoloides*) did not germinate so analysis of the whole plant, plant parts, and roots was not possible. The wild kudzu accessions as well as the *P. m. lobata* accessions all had prominent trichomes as did the commercial and *P. phaseoloides* accessions; however, the trichomes present on the *P. m. montana* accession were less pronounced. The *P. m. montana* plants also had smaller, almond shaped leaves and thinner vines as compared to the other plants (Fig. 2). The thinner vines on *P. m. montana* made the vines more malleable. The leaves of the commercial and the *P. phaseoloides* accessions were rounder than the *P. m. montana* accession. Interestingly, the leaves of the wild and *P. m. lobata* accessions tended to vary even among the same accession (Supplemental Fig. 2). While some of the *P. m. lobata* leaves were rounder, similar to that of the commercial and *P. phaseoloides* accessions, others were lobed. The lobing on the *P. m. lobata* leaves also varied from slight to deep lobing. However, irrespective of their overall shape, the leaves of the wild and *P. m. lobata* accessions tended to come to a sharp point.

### Isoflavone content

**Fig. 3:**
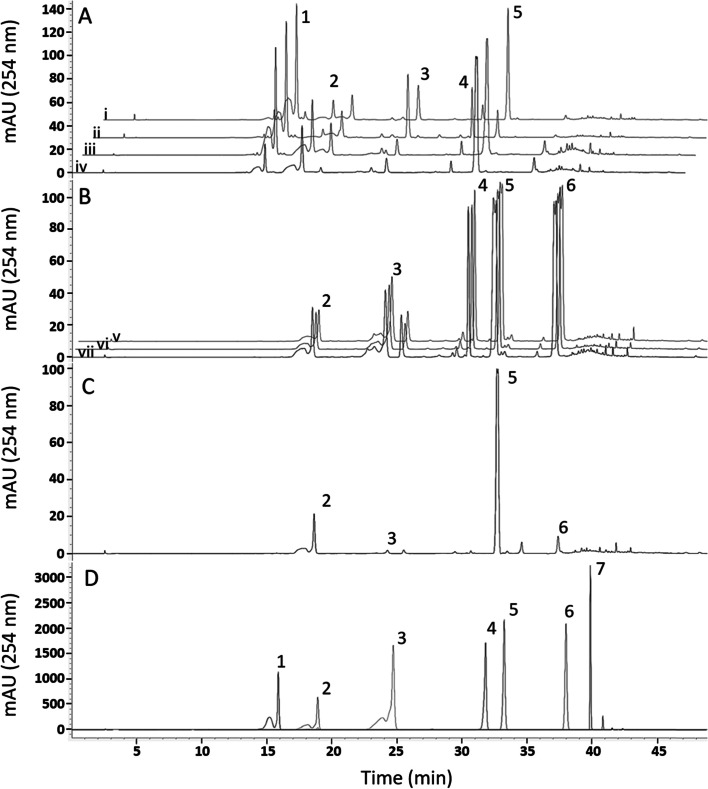
Isoflavone profiles of the roots of the eight accessions examined. **A** HPLC chromatogram showing the isoflavone profiles of the wild and *P. m. lobata* roots (a. PI 9227, b. PI 434246, c. Oklahoma, d. Texas); **B** The isoflavone profiles of the commercial and *P. phaseoloides* roots (a. Kudzu Kingdom, b. BRSEEDS, c. PI 308576); **C** The isoflavone profile of the *P. m. montana* roots (PI 298615); **D** Isoflavone standards. mAU is milli-absorbance units. 1. Puerarin, 2. Daidzin, 3. Genistin, 4. Ononin, 5. Daidzein, 6. Genistein, 7. Formononetin

An examination of the roots of all eight accessions revealed that the Oklahoma and Texas collected material and the *P. m. lobata* accessions all contained puerarin. In contrast, the commercial, *P. phaseoloides,* and *P. m. montana* accessions did not contain puerarin (Fig. 3). In addition to puerarin, roots of the wild and *P. m. lobata* accessions contained daidzin and daidzein. Other isoflavones, including genistein, genistin, and ononin were present in reduced amounts in the wild and *P. m. lobata* accessions. The commercial and *P. phaseoloides* roots contained a higher proportion of genistein, ononin, and genistin than the Oklahoma and Texas material, *P. m. lobata*, and *P. m. montana* roots. In fact, those three isoflavones were found in the highest proportion in roots of the commercial and *P. phaseoloides* accessions. The *P. m. montana* roots contained the least amount of isoflavones based on HPLC peak areas, and these were mainly daidzin and daidzein (Fig. 3C). While not containing puerarin, the commercial kudzu and *P. phaseoloides* had higher percentages of daidzein and genistein aglycones among their isoflavone complement (Supplemental Fig. 3).

### Internal transcribed spacer 2 sequencing

The internal transcribed spacer 2 (ITS2) region is generally between 200 and 250 bp. Given its small size, the entire region was able to be captured using primers from the 5.8S rRNA and 26S rRNA regions that flank the ITS2, resulting in amplicons of 425–475 bp. An Illumina MiSeq with paired end reads 2 × 300 was used, allowing for an overlap in the middle of the sequence. Following trimming and alignment, the whole sequenced amplicon was 468 bp for *P. m. lobata*, 449 bp for *P. phaseoloides*, and 436 bp for *P. m. montana.* The ITS2 region within the whole amplicon sequence was 242 bp for *P. m. lobata*, 224 bp for *P. phaseoloides*, and 211 bp for *P. m. montana*.

There were 80 nucleotide differences observed in comparisons between the *P. m. lobata* and the *P. phaseoloides* groups in the ITS2 region (Supplemental Table 1). Additional differences in the ITS2 regions were 18 nucleotide insertions/deletions (indels) in the *P. phaseoloides* group including one stretch of eight deleted nucleotides and one stretch of ten nucleotides (Supplemental Table 2). Comparisons between the *P. m. lobata* and the *P. m. montana* groups revealed 55 nucleotide differences (Supplemental Table 3) and 31 indels including one stretch of 19 deleted nucleotides in the *P. m. montana* group (Supplemental Table 4). The comparisons between *P. phaseoloides* and the *P. m. montana* groups had 51 nucleotide differences (Supplemental Table 5) and 17 indels (Supplemental Table 6).

### Maturase K (matK) sequencing

Of the ~ 1500 bp matK chloroplast gene, approximately 776 bp were amplified from the kudzu accessions using primers suggested by Yu et al. (2011) [17] for having high fidelity with angiosperms given the low nucleotide diversity found in these regions. Given the length of the amplicon to be sequenced, Sanger sequencing was used.

Following trimming and alignment of the matK sequences there were 17 single nucleotide polymorphisms (SNPs) identified between the *P. phaseoloides* and *P. m. lobata* groups, 20 SNPs identified between the *P. m. lobata* and *P. m. montana* groups, and 26 SNPs identified between the *P. phaseoloides* and *P. m. montana* groups (Table 1). Given that matK is a coding region, the amino acid substitutions that resulted from the SNPs were also examined. There were eight amino acid substitutions between the *P. phaseoloides* and *P. m. lobata* groups, 12 between the *P. m. lobata* and *P. m. montana* groups, and 15 between the *P. phaseoloides* and *P. m. montana* groups (Table 2).

**Table 1:** Maturase K (matK) SNP analysis

Position	SNP			Type
	*P. phaseoloides*	*P. m. lobata*	*P. m. montana*	
562	C	C	A	Transversion
569	C	C	G	Transversion
581	G	T	C	Variable
606	G	T	T	Transversion
706	T	T	G	Transversion
713–714	TT	GC	GC	Transversion/Transition
780	T	T	C	Transition
807	T	T	G	Transversion
810	G	T	T	Transversion
828	T	T	C	Transition
846	A	A	C	Transversion
891	G	A	A	Transition
894	T	C	C	Transition
905	C	A	A	Transversion
917	A	A	G	Transition
942	T	A	A	Transversion
948	A	G	G	Transition
954	T	G	T	Transversion
966	A	C	A	Transversion
990	G	A	G	Transition
1012	C	C	T	Transition
1014	A	C	A	Transversion
1022	C	C	T	Transition
1023	C	A	A	Transversion
1044	G	A	G	Transition
1045	C	C	A	Transversion
1073	T	T	G	Transversion
1090	A	A	C	Transversion
1098	T	A	A	Transversion
1118	C	C	T	Transition

**Table 2:** Maturase K (matK) amino acid substitutions

Position	Amino acid substitutions		
	*P. phaseoloides*	*P. m. lobata*	*P. m. montana*
188	L	L	I
190	T	T	S
194	W	L	S
202	R	S	S
236	Y	Y	D
238	L	R	R
269	N	K	K
270	E	D	D
302	S	Y	Y
306	Y	Y	C
318	H	Q	H
322	L	F	L
341	S	S	L
349	Q	Q	K
358	M	M	R
364	I	I	L
373	S	S	L

### Phylogenetic analysis

**Fig. 4:**
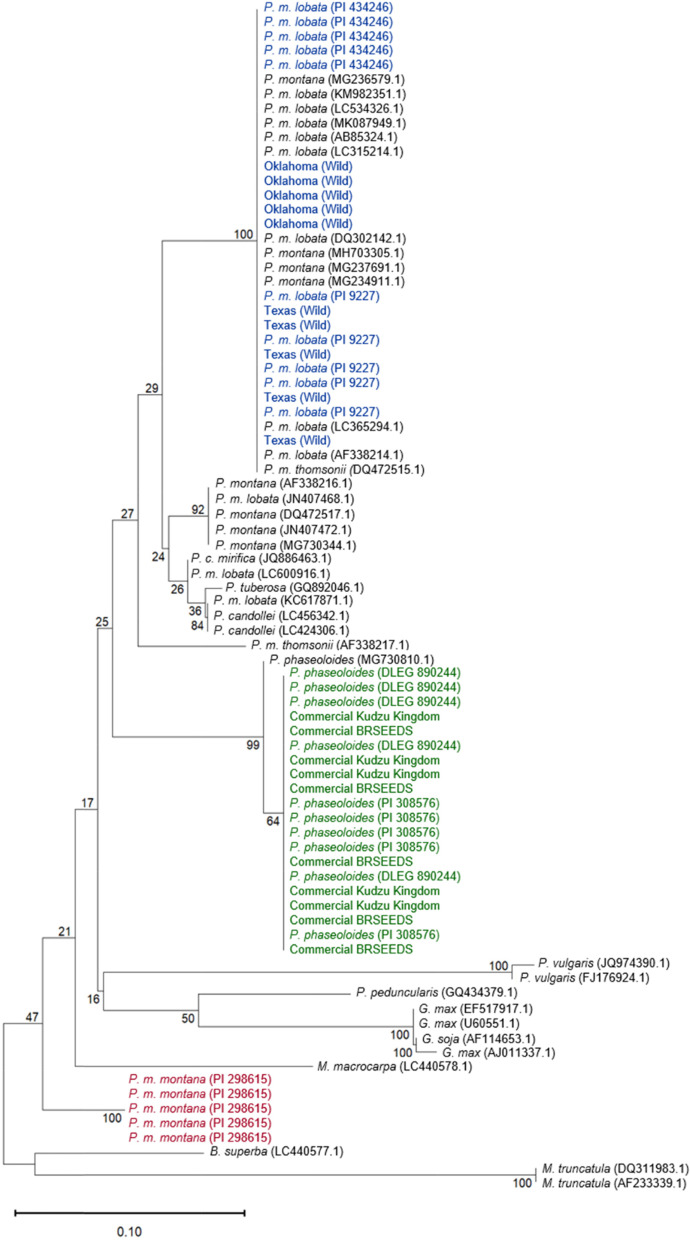
Phylogenetic tree of ITS2 sequences from the *Pueraria* accessions in the present work (colored in blue (wild and *P. m. lobata*), maroon (*P. m. montana*), and green (commercial and *P. phaseoloides*)) and those published in NCBI. The scale bar indicates the length of 0.1 substitutions. The pipeline was created using phylogeny.fr and visualized in Mega 11. (Details for pipeline in Methods)

A neighbor-joining phylogenetic tree was generated using the ITS2 and matK sequences. For the ITS2 phylogenetic tree the generated sequences were combined with sequences published in NCBI for kudzu species as well as other legumes. The results in Fig. 4 show that the *P. phaseoloides* and commercial accessions clustered together with a previously published *P. phaseoloides* ITS2 sequence from NCBI. Additionally, the *P. m. lobata* and Texas and Oklahoma ITS2 sequences clustered with *P. m. lobata* and *P. montana* sequences published at NCBI, along with a singular *P. m. thomsonii* sequence. The *P. m. montana* sequences clustered separately.

**Fig. 5:**
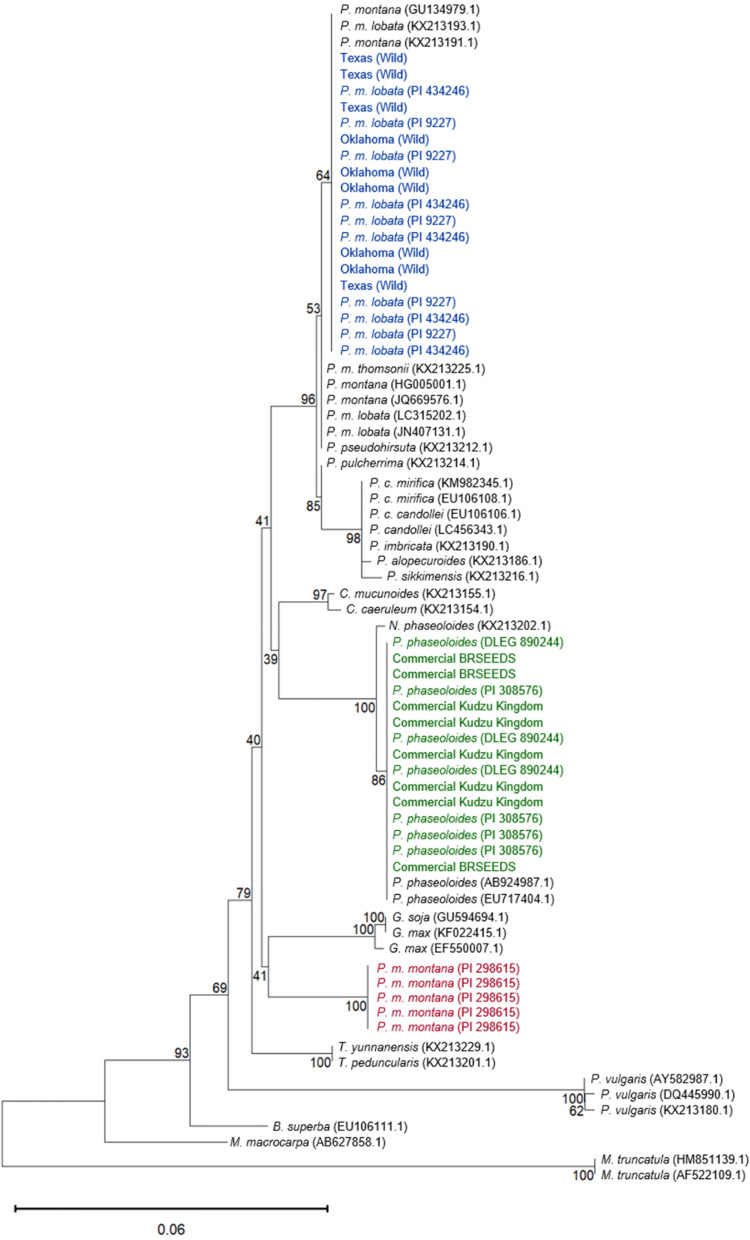
Phylogenetic tree of matK sequences from the *Pueraria* accessions in the present work (colored in blue (wild and *P. m. lobata*), maroon (*P. m. montana*), and green (commercial and *P. phaseoloides*)) and those published in NCBI. The scale bar indicates the length of 0.06 substitutions. The pipeline was created using phylogeny.fr and visualized in Mega 11. (Details for pipeline in Methods)

The phylogenetic tree for the matK sequences revealed similar clustering as the ITS2 phylogenetic tree. The *P. phaseoloides* and commercial kudzu matK sequences clustered with published matK sequences for *P. phaseoloides* and *N. phaseoloides* (formerly *P. phaseoloides*). The matK sequences of the *P. m. lobata* and Oklahoma and Texas accessions were clustered with a few *P. m. lobata* and *P. montana* sequences plus singular *P. m. thomsonii* and *P. pseudohirsuta* sequences available NCBI. However, the *P. m. lobata,* Oklahoma, and Texas kudzu matK sequences did not cluster as closely with many of the *P. m. lobata* and *P. montana* matK sequences analyzed from NCBI as they did in the ITS2 neighbor-joining tree. The *P. m. montana* matK sequences also clustered separately again, but this time they were grouped closer to other species showing more similarity to the matK sequences of *Glycine spp* (Fig. 5)*.*

### Transcriptome sequencing and assembly

To obtain *Pueraria* root transcriptomes, RNA was extracted and cDNA prepared from roots of Kudzu Kingdom (*P. phaseoloides*) and Oklahoma (*P. m. lobata*) accessions, and sequenced by the Illumina Hiseq2000 platform. The 100 bp paired-end Illumina reads were trimmed with quality scores. Clean sequence reads from *P. phaseoloides* and *P. m. lobata* were assembled separately using a combination of the programs Velvet [18] and Oases [19]. To optimize the assembly, Velvet/Oases were run with different k-mer sizes (31, 43, 55, 67, 79 and 91 nt).

Several assembly-quality parameters were assessed, including the ratio of using reads, median coverage depth, the number of contigs, the number of transcripts, the number of loci, average transcript length, and the N50 values of contigs and transcripts (Supplemental Table 7, Supplemental Fig. 4). N50 represents the sequence length L for which half of the bases in the assembly are in sequences of length N > =L. [20–22] Of the six k-mer tests in Velvet/Oases, a good balance for the above parameters was found at k-mer 55 assembly, resulting in 47,011 and 49,277 transcripts for *P. phaseoloides* and *P. m. lobata*, respectively. The full comparison of the transcriptome data for *P. phaseoloides* and *P. m. lobata* is given in Table 3.

**Table 3:** Statistics of the transcriptome data

Data	*P. phaseoloides*	*P.m. lobata*
Raw reads	38,381,722	33,214,058
Clean reads	38,014,210	32,891,280
Assembled transcripts	47,011	49,277
Percent assembled	87.8	82.9
Assembled depth	11.9	10.3
Mean length	1320	1239

To further demonstrate the quality of the assembled transcripts, the length distribution of the contigs in the two transcriptomes is shown in Supplemental Fig. 5. The N50 values of transcriptomes in *P. phaseoloides* and *P. m. lobata* were 1988 and 1881 bp, respectively. For further quality control, we mapped the assembled transcriptomes to kudzu ESTs available from GenBank (6365 ESTs) and observed that 81% (5183 ESTs) and 96% (6110 ESTs) of known EST sequences were represented in our transcriptome sets for *P. phaseoloides* and *P. m. lobata*, respectively. Kudzu ESTs were provided from a subtractive library with the *P. phaseoloides* root cDNA as the driver and *P. m. lobata* root cDNA as the target [16]. It is therefore reasonable that more kudzu ESTs are represented in the *P. m. lobata* root transcriptome set than in the *P. phaseoloides* set.

### Simple sequence repeats (SSRs) in the *Pueraria* root transcriptomes

Simple sequence repeats (SSRs) or microsatellites have been broadly used as molecular markers in marker-assisted selection for DNA fingerprinting [23, 24]. To supply SSR markers for distinguishing between *P. phaseoloides* and *P. m. lobata,* we used the MISA scripts program [25] to scan the *Pueraria* root transcriptomes to identify gene-derived SSR markers. In total, we detected 9220 and 6665 SSRs within 6729 and 5370 different transcripts from the *P. phaseoloides* and *P. m. lobata* de novo assembled transcriptomes, respectively. The putative SSRs are summarized in Supplemental Dataset 1. Excluding mono-repeats (3246 and 2625), 5974 and 4040 SSRs (dinucleotide to hexanucleotide repeats) were identified within 4516 (13.6%) and 3373 (9.7%) transcripts of *P. phaseoloides* and *P. m. lobata*, respectively. The average frequency of SSRs was one per 5.93 kb and 8.53 kb of the transcriptome sequence in *P. phaseoloides* and *P. m. lobata*, respectively. Among dinucleotide to hexanucleotide repeats, the distribution of SSRs was as follows: di- (2143, 35.9% and 1138, 28.2%); tri- (3255, 54.5% and 2606, 64.5%); tetra- (204, 0.03% and 116, 0.03%); penta- (138, 0.02% and 80, 0.02%) and hexa- (234, 0.04% and 100, 0.02%) in *P. phaseoloides* and *P. m. lobata* transcripts, respectively.

### Annotation, functional classification, mapping and quantitation of assembled transcripts

The transcriptome assembly from roots of *P. phaseoloides* and *P. m. lobata* contains 47,011 and 49,277 transcript isoforms, which represent a total of 33,221 and 34,677 distinct assembled loci, respectively. Each locus may include several highly similar transcript isoforms, such as splice variants, homologs and paralogs, and sequencing errors [16, 22]. To reduce the degree of gene redundancy, we chose the longest transcript to perform annotation as the representative of the locus.

**Fig. 6:**
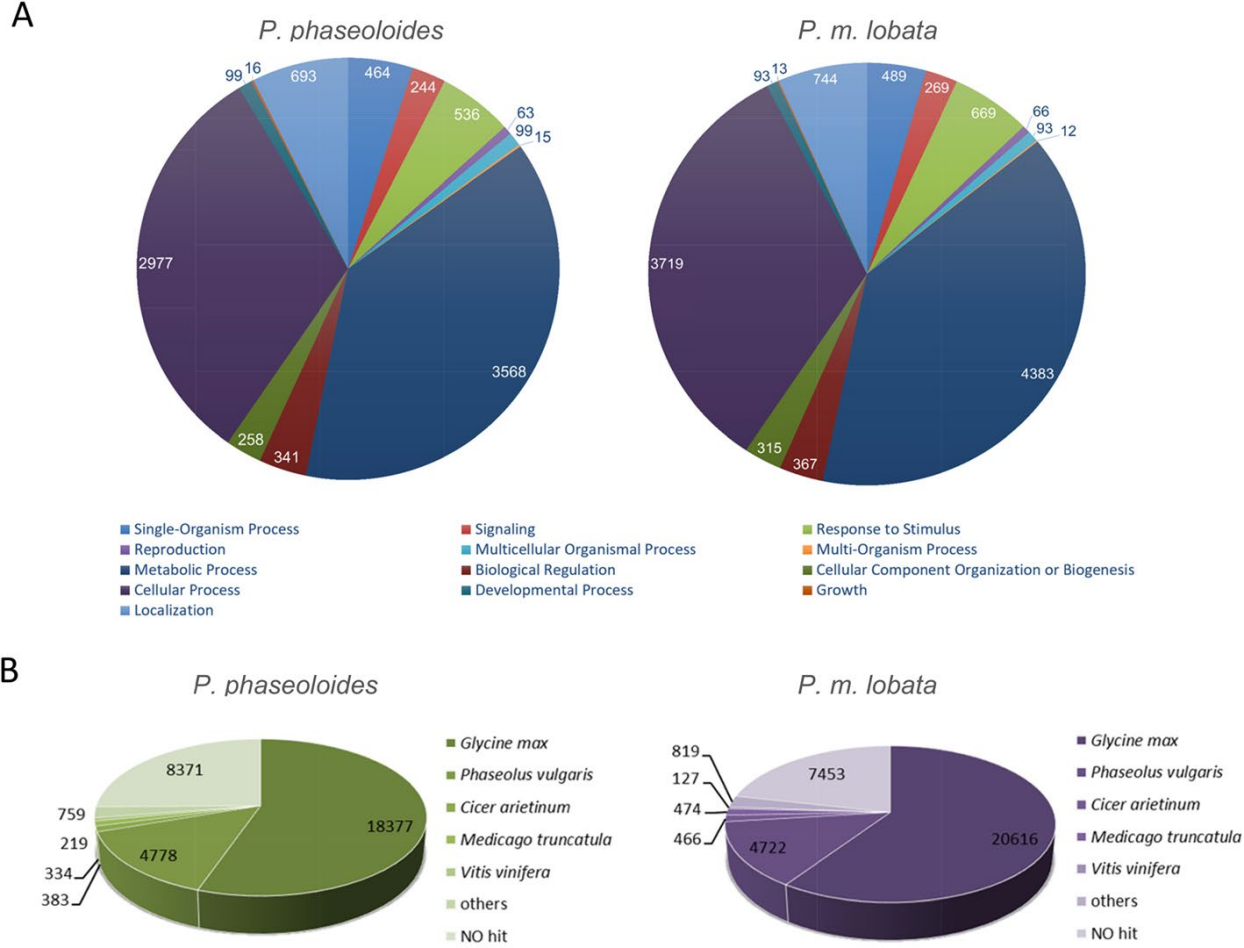
Gene ontology classification and homology characteristics of *Pueraria* root transcript sequences. **A** Gene ontology analysis of the assembled transcripts. **B** Species distribution of homology search of *Pueraria* transcriptomes against the NR database

A homology search against NR resulted in 24,850 and 27,244 annotated genes in *P. phaseoloides* and *P. m. lobata*, respectively. Among annotated genes, the most abundant genes are involved in metabolic processes according to their Gene Ontology (GO) categories using Plant GOslim ancestor terms [26–28] (Fig. 6A). Based on top hits in the NR database, *Pueraria* transcripts have strong homology to transcripts from soybean (*Glycine max*), followed by green bean (*Phaseolus vulgaris*), consistent with the close phylogenetic relationship between kudzu and soybean [29] (Fig. 6B).

**Fig. 7:**
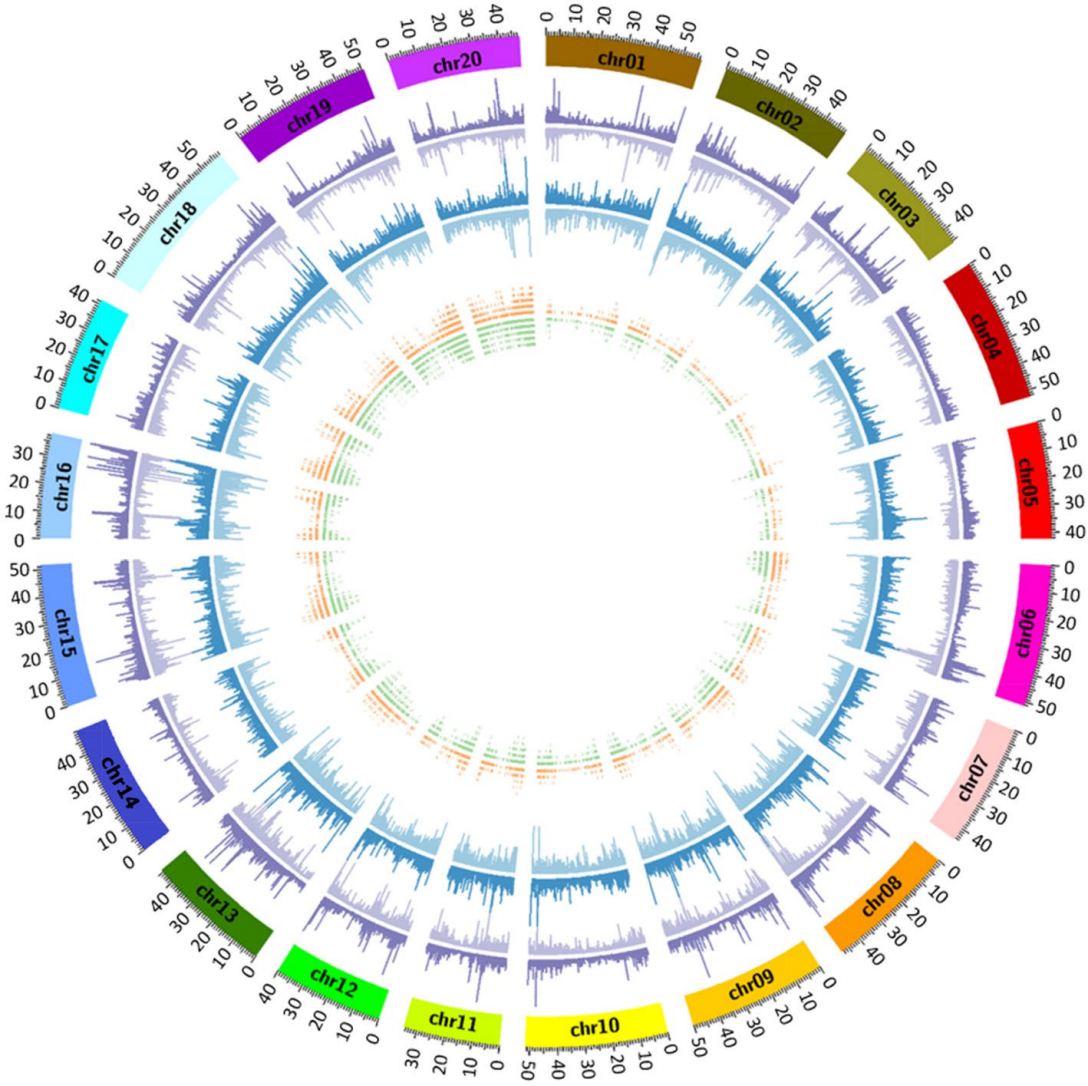
Distribution of the assembled *Pueraria* transcripts mapped to the soybean genome. External track shows the density of *P. m. lobata* transcripts aligned to the Gmax genome, in both + (outside) and – (inside) strands in purple. The middle track shows the density of *P. phaseoloides* transcripts aligned to the Gmax genome, in both + (outside) and – (inside) strands in blue. Inner track show the SSR-bearing transcripts aligned to the Gmax genome sequence, with *P. m. lobata* strands in orange (outside) and *P. phaseoloides* strands in green (inside)

To illustrate the coverage distribution of assembled transcripts on *Glycine max* as the reference genome, we aligned the transcripts to the 20 chromosomes in a 500 kb interval (Fig. 7). Both *P. phaseoloides* and *P. m. lobata* assembled transcripts covered all 20 soybean chromosomes without any large gap. The correlation between *P. phaseoloides* and *P. m. lobata* transcriptome density was 0.74, indicating genetic divergence between these two species. To pinpoint the location of polymorphisms, the SSR-bearing transcripts were uniquely anchored to the single best hit in the *Glycine max* genome. The inconsistency in the SSR locations between *P. phaseoloides* and *P. m. lobata* further indicates the genetic divergence of the two accessions.

Cross-species transcriptomic comparisons have been shown to be feasible [30, 31]. Therefore, to obtain a comparative gene expression pattern between the two *Pueraria* accessions, we aligned the sequencing reads to *Glycine max* as the reference genome [32]. Overall, 65 and 66% of the cleaned reads from *P. phaseoloides* and *P. m. lobata* were mapped to the *Glycine max* protein database, respectively, and 84% of *Glycine max* proteins were covered with at least one mapped read (Supplemental Table 8). For each Gmax protein code, the number of matching reads was counted and the hit count was then transformed to RPKM (the reads per kilobase of transcript per million) to normalize for the number of reads available for each line [30]. The coverage of the functional classes between *P. phaseoloides* and *P. m. lobata* were similar (Supplemental Fig. 6A). The majority of gene categories were well represented by more than 70% of genes in each class for both mappings. Among them, 87 and 91% of genes classified in secondary metabolism were detected in *P. phaseoloides* and *P. m. lobata*, respectively.

The average RPKM values for each accession were 19.9 and 20.3, respectively. To define “differentially expressed genes”, we used the criterion of 2-fold difference in RPKM value with the filter of RPKM value above 20 between the two RNA samples. By these criteria, 1631 and 1675 genes were considered as differentially expressed in *P. phaseoloides* and *P. m. lobata*, respectively. Overall, genes classified in photosynthesis (PS), oxidative pentose phosphate pathway (OPP), major and minor carbohydrate (CHO) metabolism, and secondary metabolism were enriched in *P. m. lobata*, whereas genes classified in C1-metabolism, S-assimilation, and DNA and RNA metabolism were more represented in *P. phaseoloides* (Supplemental Fig. 6B). A detailed comparison for genes enriched in secondary metabolism is shown in Supplemental Fig. 6C. It is clear that the transcriptome of *P. m. lobata* is enriched in genes encoding proteins involved in flavonoid biosynthesis.

## Discussion

### Identification of kudzu species using barcoding

With the verified samples provided by GRIN-Global, the wild collected and commercial kudzu accessions compared previously for puerarin production [16] were identified as *P. montana lobata* and *P. phaseoloides*, respectively. The ITS2 and matK sequences for the *P. m. lobata* and Oklahoma kudzu accessions matched one another and had clear differences from the *P. phaseoloides* and commercial kudzus, which also matched one another, and had clear differences from the *P. m. montana* kudzu. The seed morphology of the *P. m. montana* and *P. phaseoloides* was most similar in shape while the seeds of *P. m. lobata* and *P. phaseoloides* were most similar in size. The plant morphology of the *P. m. lobata* and the *P. phaseoloides* was most similar with thicker vines and larger leaves. The *P. m. lobata* and wild kudzu accessions were the only plants analyzed that contained puerarin. The puerarin content for these accessions is consistent with previous reports [33].

The use of ITS2 and matK combined proved beneficial in strengthening the identification of the different *Pueraria* species. Although the ITS2 region analyzed was smaller than the matK region analyzed, there were more nucleotide differences found in the ITS2 region, presumably because it is a non-coding region. The ITS2 region varied in size for all three kudzu species analyzed, from 211 bp to 242 bp. The primers used included a plant-specific forward primer located in the 5.8S RNA and a universal reverse primer located in the 26S RNA. The plant-specific forward primer offers benefits by reducing the unintended amplification of other organisms such as fungi. Using the primers in the 5.8S and 26S regions resulted in an amplicon size between 450 and 500 bp. This amplicon size was perfect for using next generation sequencing (NGS). The use of NGS helps reduce noise that can be generated from amplification and sequencing bias by allowing for greater depth of coverage. The greater coverage depth also allows for any incorrect sequences to be muffled by the true sequence. This noise was further reduced by using low cycle numbers in the amplification prior to sequencing. The difference in size can make alignment difficult; however, using primers in the relatively conserved 5.8S and 26S regions helps overcome alignment and amplification problems [34]. In contrast, despite the reduced number of nucleotide changes, the matK region aligned perfectly across all three species analyzed. The ease of alignment for matK is common given that it is a coding region of the chloroplast [17].

The use of published ITS2 and matK sequences from other plants and *Pueraria* species in a neighbor-joining tree with the sequences generated showed clear clustering of the *P. phaseoloides* and commercial kudzus with *P. phaseoloides* plants published in NCBI. The *P. m. lobata* and wild-collected Oklahoma and Texas kudzu clustered with the other *P. m. lobata* and *P. montana* sequences while the *P. m. montana* sequences clustered separately. The neighbor-joining trees for both genes resulted in similar clades encompassing the different accessions analyzed along with the published sequences in NCBI. Although a single concatenated tree that included both genes could have provided additional resolving power to show the relatedness of all the accessions analyzed, there was a lack of ITS2 and matK sequences in NCBI from the same samples of kudzu and other legumes, making such analysis not possible.

Unlike with animals where the cytochrome oxidase I (COI) gene of the mitochondria is considered the gold standard for species differentiation, plants do not currently have a specific region that is accepted as having good discriminatory value. However, several regions have been proposed as well as the use of two regions together [35, 36]. The ITS2 region has been shown to have high discriminatory power in both Fabaceae genera and angiosperms [37–43]. In *Vigna *species, coupling matK and ITS2 increased the resolving power of the barcodes compared to using them individually [40].

The use of the ITS2 and matK regions can successfully differentiate species of the genus *Pueraria* as well as variants of the same species. The ITS2 and matK for *P. m. lobata* and *P. phaseoloides* were generated from four different populations of the respective species. The sequences for the populations of each species matched one another as well as from samples within the populations. This shows that for the kudzu species analyzed, ITS2 and matK have enough nucleotide exchange to differentiate the different species but do not segregate out different populations of the same species. The ability of these two regions to not set apart different populations of the same species is extremely important in allowing for clear identification of kudzu species regardless of where the plant originated. The ITS2 and matK sequences generated have been uploaded to BOLD (Barcode of Life Database) [44] to be available to other researchers attempting to identify plants whether directly or through the examination of plant material present in supplements or even in the feces of organisms to understand their diet as done by Yamamoto and Uchida (2018) [12].

Interestingly, the seed and plant morphology, barcoding sequence differences, and phylogenetic separation between *P. montana montana* and *P. montana lobata* would suggest that these plants are more than mere varieties of the same species as suggested by van der Maesen [8]. The differences present at both a phenotypic and genotypic level for these plants align with their being separate species as previously suggested by Ohashi et al. [45]. Ohashi et al. suggest the presence of two species, *P. montana* and *P. lobata,* where *P. lobata* has the subspecies *P. l. lobata* and *P. l. thomsonii*. A comprehensive analysis of *P. l. thomsonii* (also known as *P. thomsonii* and *P. m. chinensis*) as done here for *P. m. lobata*, *P. m. montana*, and *P. phaseoloides* could discern whether *P. l. thomsonii* is best categorized as a subspecies of *Pueraria lobata* or as its own species.

### Summary of the transcriptome dataset

The rapid development of next-generation sequencing (NGS) technologies has enabled discovery of novel genes by using the RNA-seq approach [46, 47]. To provide a basis for a better understanding of the bioactive natural products in kudzu, we have performed a comparative whole root transcriptome analysis. Three other reports have generated transcriptomes for different tissue types of *P. m. lobata* [48–50], and more recently, for different tissues of *P. thomsonii* and *P. candollei* var. *mirifica* [51, 52]. However, none of these analyses examine two different kudzu species for comparative gene expression. A previous phylogenetic study showed 80% of US kudzu analyzed had matching genotypes with one or more samples from the same population [53]. This suggest that the transcriptome generated from kudzu from Oklahoma (*P. m. lobata*) could be a representative genomic resource for this noxious weed that dominates throughout the Southeastern US. In Oklahoma alone a report suggests a loss of almost $168 million in the lumber industry over 5 years [54]. Knowledge of its transcriptome can lead to development of methods of biological eradication.

It is challenging to perform de novo assembly of transcriptomes in non-model organisms lacking a reference genome. Early studies demonstrated that optimization of the transcriptome assembly using various k-mer lengths is highly desirable for de novo assemblies [22, 55, 56]. In the present study, various parameters were analyzed with a combination of Velvet and Oases. Velvet/Oases start by constructing de Bruijn graphs directly from sequencing reads, remove errors, and then resolve each de Bruijn graph to extract transcripts for each connected component (called “loci”) in the graph [18, 19, 22]. Velvet/Oases allow a range of k-mer sizes to accommodate variation in read coverages among genes. Longer k-mers lead to more specificity, with lower coverage and sensitivity. Assembly quality decreases towards both lower and higher k values [18, 19, 22]. Assembly quality tests were performed to determine the most suitable parameter; the usage ratio of reads, depth, length, and number of assembled transcripts [22, 55, 56]. The Velvet/Oases k–mer 55 assembly was selected as the representative for the *Pueraria* root transcriptomes, resulting in 47,011 and 49,277 transcripts with 33,221 and 34,677 loci, respectively. This is consistent with the gene number for the majority of sequenced plant genomes of between 20,000 and 40,000 [21].

### Differentiation of *Pueraria* species

Simple sequence repeats (SSRs) markers have been widely used in plant genetic studies because of their tendency toward being multiallelic, expression of both parental alleles, quantity, and vast coverage in genomes [57]. Genic SSRs (derived from genes, ESTs, or cDNA clones) have some advantages over genomic SSRs including being easily generated, characterized, and possessing transferability between different species [58].

Previous markers identified to distinguish kudzus included 13 allozyme loci, 11–49 randomly amplified polymorphic DNAs (RAPDs), and 13–15 microsatellite locations [9, 53, 59–62]. Most recently, genic SSRs were identified from *P. m. montana* and *P. phaseoloides* [63]. Some of these reports used other kudzu species or varieties; however, the goal of all of them was beyond identification and focused more on population/genetic diversity and origin of kudzu’s introduction. Here we identified 9220 and 6665 genic SSRs from the assembled transcripts from *P. phaseoloides* and *P. m. lobata*, respectively. Excluding mono-SSRs, 5974 and 4040 genic SSRs were detected in 13.6 and 9.7% of the transcripts with the frequency of one SSR per 5.93 kb and 8.53 kb in the *P. phaseoloides* and *P. m. lobata* transcriptomes, respectively. Frequencies of genic SSRs were reported as 1 per 3.92 kb or 8.63 kb from de novo assembled transcriptomes in the legume species lentil and chickpea, respectively [56, 64]. Additionally, the genic SSR frequency in Chinese sweetgum was 1 per 5.12 kb [65].

Factors affecting the frequency and types of SSRs include the taxon, the genomic make-up, and the SSR mining length used for analysis [66]. Here we applied the same parameters for mining microsatellites in the *P. phaseoloides* and *P. m. lobata* transcriptomes, so the differences in SSR frequency likely indicate differences in genomic composition. Except for mono-repeats, the most abundant SSRs were tri-nucleotide repeats (54.5 and 64.5%), then di-nucleotide repeats (35.95 and 28.2%) in *P. phaseoloides* and *P. m. lobata* transcripts, respectively. This is consistent with the observation that tri-SSRs are generally the most frequently occurring SSRs found in genic SSRs, followed by di-SSRs [58, 67]; however, there are exceptions as with *Camellia japonica* [68]. Among all the tri-nucleotides, AAG/CTT was found to be the most frequent motif, consistent with recent studies [69–71]. Our results suggest that the SSRs identified here are reliable and can be useful tools for assaying genetic variation in *Pueraria* populations.

Cross-species mapping in protein space is a viable strategy to compare different species when an equidistant reference is available [30]. Through mapping reads by alignment on the soybean protein sequence, we quantified transcript abundance in *P. phaseoloides* and *P. m. lobata.* Transcripts catalogued in photosynthesis, major CHO metabolism and minor CHO metabolism were enriched in the wild-collected, invasive *P. m. lobata* compared with the commercial species *P. phaseoloides.* This is consistent with the competitive ability of *P.m. lobata* for fixing carbon [72]. Transcripts classified in secondary metabolism were also enriched in *P. m. lobata,* particularly genes involved in flavonoid biosynthesis*.*

## Conclusions

Puerarin is found in some but not all species of *Pueraria*. Here we have identified the ITS2 and matK barcodes as sufficient to differentiate between three kudzu species (*P. montana*, *P. lobata*, and *P. phaseoloides*), and in so doing identified the wild and commercial kudzu species used previously for preliminary gene identification in the puerarin pathway [16]. We have also provided molecular tools for more in-depth differential expression analysis of natural product pathways between transcriptomes of *P. m. lobata* and *P. phaseoloides*, as well as the identification of microsatellites for further use to aid in identification of the two species.

## Methods

### Chemicals

Daidzin, genistein, and genistin were purchased from Cayman Chemical Company (Ann Arbor, MI). All other standards were purchased from Indofine Chemical Company (Hillsborough, NJ). HPLC solvents were from FisherSci (Walthanm, MA). Other chemicals were purchased from Sigma-Aldrich (St. Louis, MO) unless otherwise indicated.

### Seeds

Oklahoma wild kudzu seeds were collected (under Texas Department of Agriculture permit no 14-NIPP-01) from P street SE, near the junction with Springdale Road, in Ardmore, OK (34.159, − 97.108). The kudzu from Oklahoma had previously been identified as *P. montana* [73]. Kudzu Kingdom seeds were ordered from Kudzu Kingdom, a division of SunTop Inc., in Kodak, TN. Texas wild kudzu seeds were collected (under Texas Department of Agriculture permit no 19-NIPP-01) off Copeland road under Batman the ride at Six Flags Over Texas in Arlington, TX (32.759, − 97.067). The kudzu from Texas had previously been identified as *P. m. lobata* and validated by Texas Invaders (Site Record 19,737). BR seeds were ordered from the company BRSeeds in Araçatuba, São Paulo, Brazil as *P. phaseoloides*. *P. montana* (Lour.) Merr. var. *lobata* (Willd.) collected in the United States (PI 434246); *P. montana* (Lour.) Merr. var. *lobata* (Willd.) collected in Kanagawa, Japan (PI 9227); *P. montana* (Lour.) Merr. var. *montana* donated from Taiwan (PI 298615); *Neustanthus phaseoloides* (Roxb.) Benth. (formerly *P. phaseoloides* (Roxb.) Benth.) collected in Venezuela (PI 308576) were ordered through USDA Grin Global from the Plant Genetic Resources Conservation Unit in Griffin, GA (under Texas Department of Agriculture permit no 19-NIPP-01 where applicable). *N. phaseoloides* (DLEG 890244) seeds collected from an unknown location were ordered through USDA Grin Global from the Desert Legume Program in Tucson, AZ. Seeds ordered through USDA Grin Global were verified by an ARS Systematic Botanist and are publicly available.

### Seed sterilization, germination, and plant growth conditions

Seeds were scarified in sulfuric acid for 20 min (BR seeds, Kudzu Kingdom seeds, USDA *P. phaseoloides*, and USDA *P. montana var. montana* seeds), or 45 min (Texas, Oklahoma, and USDA *P. montana lobata* (Origins Japan and US). They were then rinsed with copious amounts of water three times, dried and sterilized in 20% (v/v) bleach for 5 min. The seeds were allowed to dry before being plated on water agar. The plates were placed in the dark at 4 °C for 5 days, then moved to a 24 °C light chamber and monitored for germination. Once germinated the seeds were placed in a greenhouse with temperature settings from 20 °C–28 °C and at least 14 h of light.

For root isoflavone analysis a young vine was cut from the main plant and the cut tip dipped in IBA (indole 3-butyric acid) before being placed in damp soil. The cuttings were monitored and after 4 weeks were repotted. After 8 weeks the roots were washed of excess soil and harvested for isoflavone analysis.

### DNA isolation

Tissues, including leaves and seeds, were collected and placed in 2 mL Eppendorf tubes with a single ball bearing. The tubes were placed in liquid nitrogen and the tissue was ground using a Retsch Mixer Mill 400 at 30 Hz for 15 s. The samples were then checked for degree of grinding and placed in liquid nitrogen. If the tissue was not thoroughly ground, it was run on the Retsch Mill again until efficient tissue grinding was achieved.

Tissue was suspended in 500 μL of 2X CTAB extraction buffer, vortexed for 5 s to mix and placed in a 60 °C oven for 30 min with occasional mixing. Tissue was centrifuged at room temperature at 16,000 x g for 5 min. The upper liquid was transferred to a new tube being careful to avoid the tissue debris. An equal volume of cold chloroform was added to the tubes, which were then vortexed for 5 s and centrifuged at 4 °C for 10 min at 12,000 x g. The upper aqueous phase was carefully transferred to a new tube, an equal volume of cold chloroform was added, the mixture vortexed for 5 s and then centrifuged at 4 °C for 10 min at 12,000 x g. The upper aqueous phase was collected, an equal volume of cold isopropanol was added, the tube incubated at room temperature for 10 min, and then centrifuged at 4 °C for 10 min at 12,000 x g. The liquid was carefully poured off and 1 mL of 70% (v/v) ethanol was added to the tube, which was centrifuged for 1 min at room temperature at 12,000 x g. The liquid was again poured off, the tube re-centrifuged for 10 s and the remaining liquid carefully removed avoiding the pellet. The tube was briefly placed in a centrifuge with a cold trap (SpeedVac) to remove any residual ethanol. The pellet was resuspended in 50 μL ddH2O. The DNA concentration was calculated on a NanoDrop™ 2000.

### Flavonoid extraction

Root tissue was collected from plants and placed in a 2 mL Eppendorf tube with a single ball bearing. The tissue was placed in liquid nitrogen before being lyophilized on a Labconco freeze dryer for 3 days. The tube was then placed in liquid nitrogen and ground on a Retsch Mixer Mill 400 at 30 Hz for 15 s. Twenty mg of tissue was transferred to a new tube and remaining tissue stored at -80 °C. The 20 mg of tissue was resuspended in 1.5 mL of 80% (v/v) methanol and sonicated for 1 h in an ice water ultrasonic bath (Branson, Danbury, CT). Following sonication, the tubes were placed on an end-over-end rotator at 4 °C overnight, then centrifuged for 20 min at 12,000 x g. The supernatant was transferred to a new tube being careful to avoid the tissue debris pelleted at the bottom of the tube. The tubes were placed on a nitrogen evaporator (Organomation Associates Inc., Berlin, MA) to dry under a stream of air/nitrogen. After the contents of the tubes had dried, 250 μL of ddH2O was added and the tubes placed on an end-over-end rotor at 4 °C for 1 h.

Ethyl acetate extraction of flavonoids was performed twice by adding 2 times the volume of ethyl acetate to the tube, inverting to mix, and centrifuging at 12,000 x g for 10 min at 4 °C. The top layer was transferred to a new tube and dried under a stream of air/nitrogen on an Organomation nitrogen evaporator. The contents of the tubes were resuspended in 150 μL of 100% methanol. The samples were then analyzed by HPLC.

### ITS2 metagenomic sequencing

The ITS2 region was sequenced in collaboration with the BioDiscovery Institute (BDI) Genomics Center (Denton, TX) and Salient Genomics LLC (Krum, TX). Total DNA was used to amplify the ITS2 regions with barcode and index adapters attached to ITS2 primer sequences ITS-p3/ITS-u4 [34]. The samples were prepped and run on an Illumina MiSeq (Illumina, Inc., San Diego, CA). Prior to sequencing, DNA from every accession was amplified with the ITS-p3/ITS-u4 primers to check amplicon size [34]. When run on a 1% agarose gel, all of the amplicons ran just under the 500 bp band of the ladder, consistent with the expected amplicon size of around 450 bp. However, the size of the *P. m. montana* amplicon was slightly lower than that of the other accessions consistent with the sequencing results.

### matK Sanger sequencing

DNA samples were amplified with matK primers [17] using NEB’s Q5 Hot-start polymerase following the manufacturer’s instructions including extension time. The annealing temperature was calculated using NEB’s Tm calculator. Following amplification, the samples were sent to Eurofins Genomics (Louisville, KY) for PCR clean-up and one-pass Sanger method sequencing. To confirm the amplicons prior to sequencing, they were run on a 1% agarose gel. All the amplicons ran between the 500 bp and 1000 bp band of the ladder, consistent with the expected amplicon length of around 775 bp.

### Barcode sequence analysis

Barcoding sequences were analyzed using Geneious Prime (San Diego, CA). Once the sequences were imported in Geneious Prime they were paired and trimmed using the BBDuk plugin to remove Illumina adapters as well as low quality (below 30) and short (less than 100 bp) reads (for ITS2 sequences). The forward and reversed reads were merged together using BBMerge. Merged sequences with a length between 430 and 480 bp were extracted (for ITS2 sequences). The reads were assembled de novo using the Geneious assembler and a consensus sequence was generated for each sample. The samples were aligned for each amplicon group to identify SNPs and Indels between the three accessions.

### Barcoding phylogenetic trees

The phylogenetic trees were made using a pipeline built with phylogeny.fr. The pipeline settings used MUSCLE for the sequence alignment, Gblocks for the alignment curation, and ProtDist/FastDist + BioNJ for building the phylogenetic tree with a bootstrap value of 1000. The phylogenetic tree was viewed and edited with Mega 11 [74–81].

### HPLC analysis

Twenty μL samples were injected on an Agilent 1220 Infinity II with a C18 reverse phase column. The 50 min run used the solvents 0.1% (v/v) formic acid (A) and acetonitrile (B) with a gradient as follows: 0–5 min, 95% A; 5–10 min, 85% A; 10–25 min, 77% A; 25–30 min, 67% A; 30–35 min, 60% A; 35–40 min, 0% A; 40–45 min, 0% A; 45–50 min, 95% A with a flow rate of 1 mL/ min. Absorption was measured at 254 nm.

### RNA extraction, cDNA library construction and Illumina sequencing

As described [82], each RNA-library was prepared from 1 μg of total RNA isolated from one sample each of Kudzu Kingdom (*P. phaseoloides*) and Oklahoma (*P.m. lobata*) roots using TruSeq RNA Sample Prep Kits v2 (Illumina Inc., San Diego, CA), according to the manufacturer’s instructions, at the Genomics Core Facility at the Noble Foundation. The prepped samples with individual indexes were pooled together to run on one Hiseq2000 lane targeting 100 bp paired reads. The Hiseq2000 run was conducted at the Genomics Core Facility of the Oklahoma Medical Research Foundation, Oklahoma City.

### Short read de novo assembly of transcriptomes

Processing of the 100 bp paired-end Illumina reads began by interleaving the read mates for each sample into a single file and trimming bases with quality scores of 20 or less from the end of each read. Reads less than 40 bp long after trimming were discarded along with their mates [82]. Each of the *Pueraria* root Illumina libraries was assembled separately using a combination of Velvet 1.2.10 [18] and Oases 0.2.08 [19]. To optimize the assembly towards higher contiguity and specificity, Velvet was run using different hash lengths (k-mers 31, 43, 55, 67, 79 and 91) with an average insert length of 300 bp. The results of the Velvet assemblies were then run through Oases using an insert length of 300 bp. Other parameters of Velvet and Oases were set as default.

### Annotation

The assembled transcript isoforms were searched against the NCBI NR database using blastx alignment (1e-6) [83], and further annotated with default parameter values using Blast2Go [84]. After the Blast2Go mapping process, EC numbers from the KEGG pathway [85] and GO terms were generated.

### SSR detection

In a pre-process step, poly-T (poly-A) stretches from the 5′ (3′) were removed by EST-trimmer scripts [86]. Parameters were set as removing (T)5 or (A)5 in a range of 50 bp on the 5′- or 3′-end, respectively. Sequences of less than 100 bp were discarded and sequences larger than 3000 bp were clipped at their 3′ side [30]. Then trimmed sequences were analyzed using MISA scripts [30] to identify Simple Sequence Repeats (SSRs). Mono-, di, tri-, tetra-, penta- and hexanucleotide repeats with a minimum of 10, 7, 5, 5, 5, and 5 subunits were regarded as SSRs, respectively.

### Mapping and quantification of sequence reads

As described [30], the Illumina sequence reads were mapped onto coding sequences of the *Glycine max* genome (version Gmax_275_Wm82.a2.v1 download from Phytozome website) by blastx [83] with threshold as 1e-6. To reduce multiple-mapping problems, coding sequences from primary transcripts without alternative splice sites in the *Glycine max* genome were used [32]. The blastx output was parsed with in-house PERL scripts to count the number of reads mapped to each Glymax protein and then to calculate the RPKM value for every Glymax protein in each library.

## Supplementary Information

**Additional file 1: Supplemental Figure 1.** Images of vines and whole plant morphology. **Supplemental Figure 2.** Leaves from USDA PI 9227 *P. m. lobata* plants. **Supplemental Figure 3.** Percent composition of six common isoflavones in each of the seven accessions. **Supplemental Figure 4.** Quality measurements for Velvet/Oases assemblies. **Supplemental Figure 5.** Length distribution of the assembled transcripts in *P. phaseoloides* and *P. m. lobata*. **Supplemental Figure 6.** Pathway representation analysis of the soybean transcripts mapped by *Pueraria* reads. **Supplemental Table 1.** ITS2 nucleotide changes between *P. m. lobata and P. phaseoloides*. **Supplemental Table 2.** ITS2 insertions/deletions between *P. m. lobata* and *P. phaseoloides*. **Supplemental Table 3.** ITS2 nucleotide changes between *P. m. lobata* and *P. m. montana*. **Supplemental Table 4.** ITS2 insertions/deletions between *P. m. lobata* and *P. m. montana*. **Supplemental Table 5.** ITS2 nucleotide changes between *P. phaseoloides* and *P. m. montana*. **Supplemental Table 6.** ITS2 insertions/deletions between *P. phaseoloides* and *P. m. montana*. **Supplemental Table 7.** Assembly statistics (Velvet/Oases) for *P. phaseoloides and P. m. lobata.* **Supplemental Table 8.** Statistics of *Pueraria* reads mapped to soybean by BLAST.

**Additional file 2: Supplemental Dataset 1.** Putative SSRs from transcripts of *P. phaseoloides* and *P. m. lobata.*

## Data Availability

The DNA barcoding sequences are available on the BOLD system with the processIDs KUDZU002–21 to KUDZU046–21. Sequence data from this article can be found in the NCBI Sequence Read Archive (SRA) repository, NCBI SRA accession No. SRX768865. The assembled transcriptomes can be found at NCBI, accession numbers 10672212 and 10671973.

## Abbreviations

BLAST: Basic local alignment search tool
BOLD: Barcode of life database
CHO: Carbohydrate
COI: Cytochrome oxidase I
CTAB: Cetyltrimethylammonium bromide
DNA: Deoxyribonucleic acid
EC: Enzyme nomenclature
EST: Expressed sequence tag
GO: Gene ontology
GPS: Global positioning system
GRIN: Germplasm resource information network
HPLC: High performance liquid chromatography
IBA: Indole 3-butyric acid
ITS: Internal transcribed spacer
ITS2: Internal transcribed spacer 2
KEGG: Kyoto encyclopedia of genes and genomes
matK: Maturase K
mAU: Milli-absorbance units
MISA: Microsatellite identification tool
NCBI: National center for biotechnology information
NGS: Next generation sequencing
NR: Non-redundant protein
OPP: Oxidative pentose phosphate
PCR: Polymerase chain reaction
PERL: Practical extraction and reporting language
PS: Photosynthesis
RNA: Ribonucleic acid
RPKM: Reads per kilobase of transcript per million
SNP: Single nucleotide polymorphism
SRA: Sequence read archive
SSR: Simple sequence repeat
USDA: United States Department of Agriculture
