# Supplementary material for: Identification of Pueraria spp. through DNA barcoding and comparative transcriptomics
Source: BMC Plant Biol. 2022 Jan 3;22:10. doi: 10.1186/s12870-021-03383-x (PMC8722073; doi:10.1186/s12870-021-03383-x)
Supplement: Supplementary file 1 — Additional file 1: Supplemental Figure 1. Images of vines and whole plant morphology. Supplemental Figure 2. Leaves from USDA PI 9227 P. m. lobata plants. Supplemental Figure 3. Percent composition of six common isoflavones in each of the seven accessions. Supplemental Figure 4. Quality measurements for Velvet/Oases assemblies. Supplemental Figure 5. Length distribution of the assembled transcripts in P. phaseoloides and P. m. lobata. Supplemental Figure 6. Pathway representation analysis of the soybean transcripts mapped by Pueraria reads. Supplemental Table 1. ITS2 nucleotide changes between P. m. lobata and P. phaseoloides. Supplemental Table 2. ITS2 insertions/deletions between P. m. lobata and P. phaseoloides. Supplemental Table 3. ITS2 nucleotide changes between P. m. lobata and P. m. montana. Supplemental Table 4. ITS2 insertions/deletions between P. m. lobata and P. m. montana. Supplemental Table 5. ITS2 nucleotide changes between P. phaseoloides and P. m. montana. Supplemental Table 6. ITS2 insertions/deletions between P. phaseoloides and P. m. montana. Supplemental Table 7. Assembly statistics (Velvet/Oases) for P. phaseoloides and P. m. lobata. Supplemental Table 8. Statistics of Pueraria reads mapped to soybean by BLAST. [file 12870_2021_3383_MOESM1_ESM.docx]

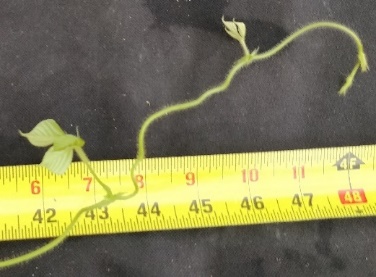

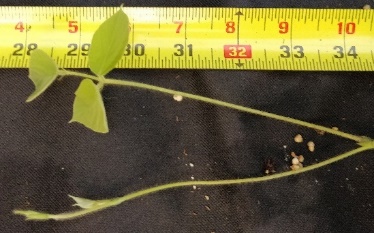

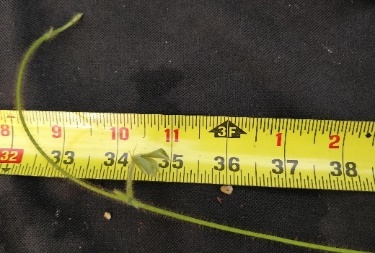

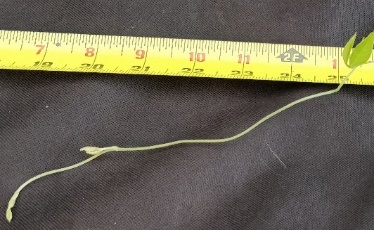

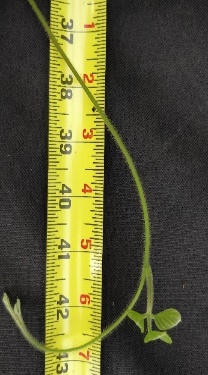

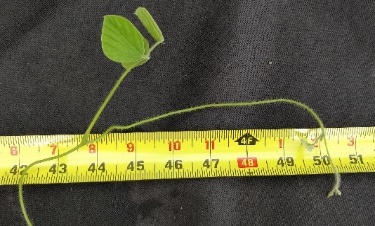

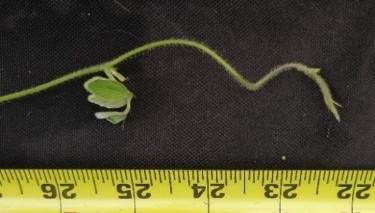

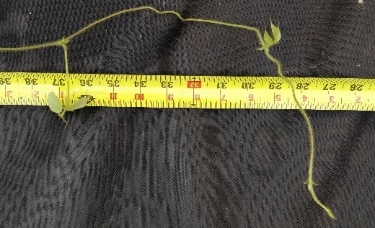


A

C

E

G

I

K

M

O


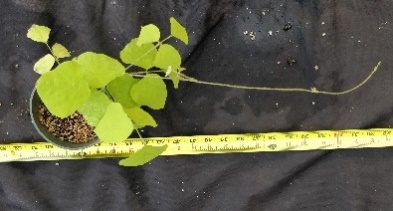

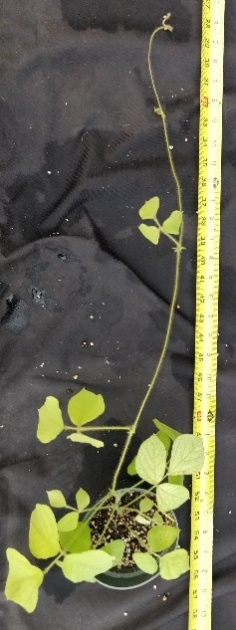

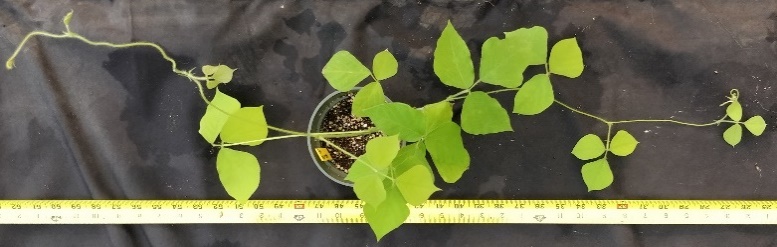

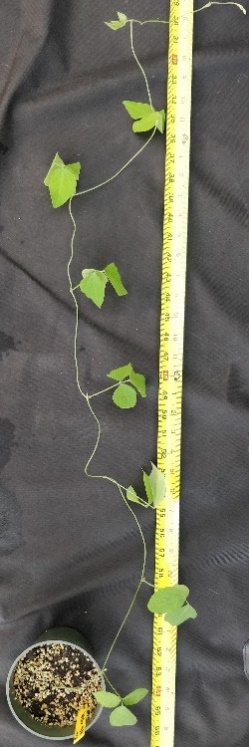

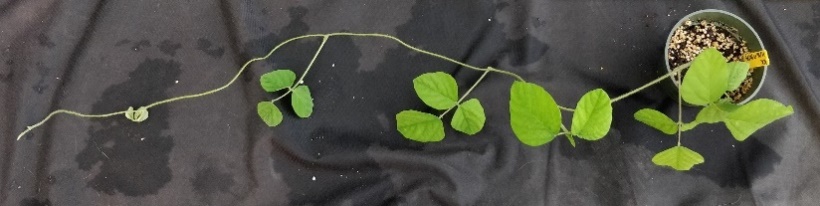

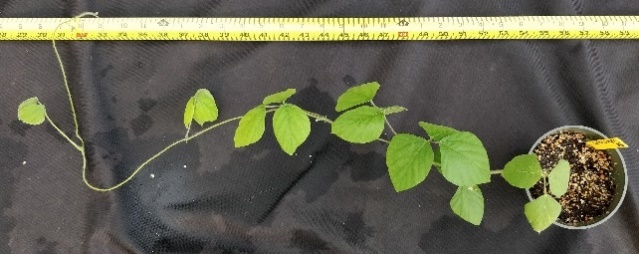

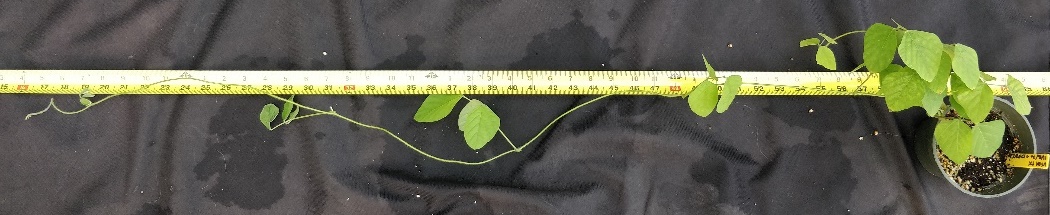

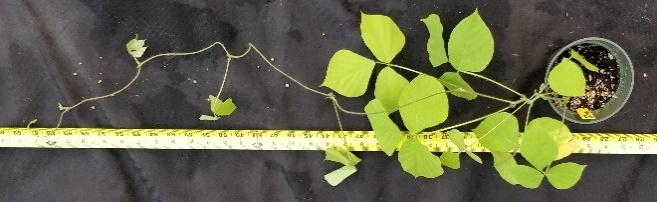


B

D

F

H

J

L

N

P

**Supplemental Figure 1**. Images of vines and whole plant morphology. A-B. Oklahoma (wild); C-D. Texas (wild); E-F. PI 9227 (*P. m. lobata*); G-H. PI 434246 (*P. m. lobata*); I-J. PI 298615 (*P. m. montana*); K-L. Kudzu Kingdom (commercial); M-N. BRSEEDS (commercial); O-P. PI 308576 (*P. phaseoloides*)


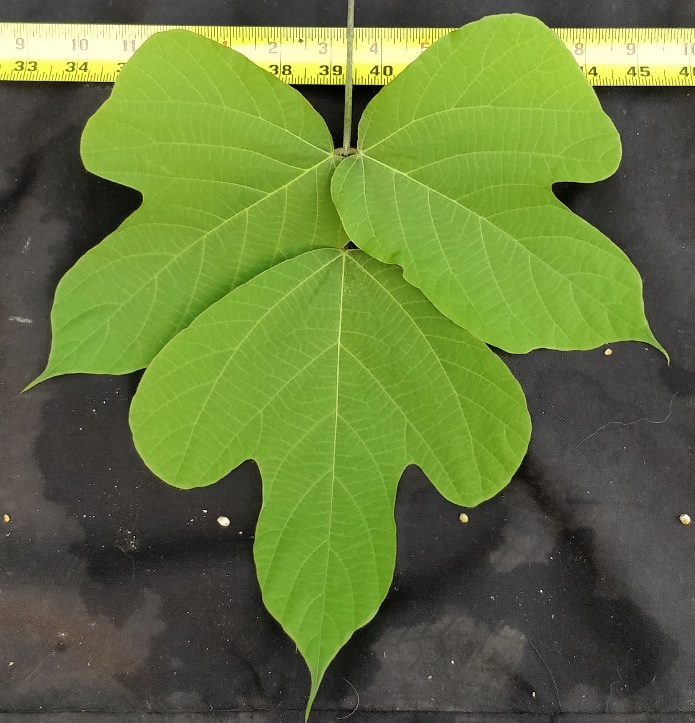

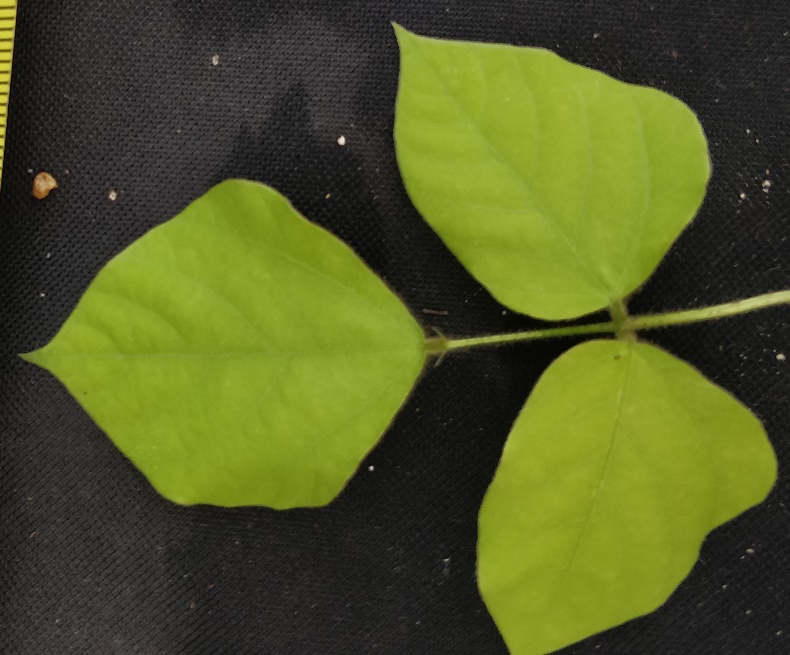

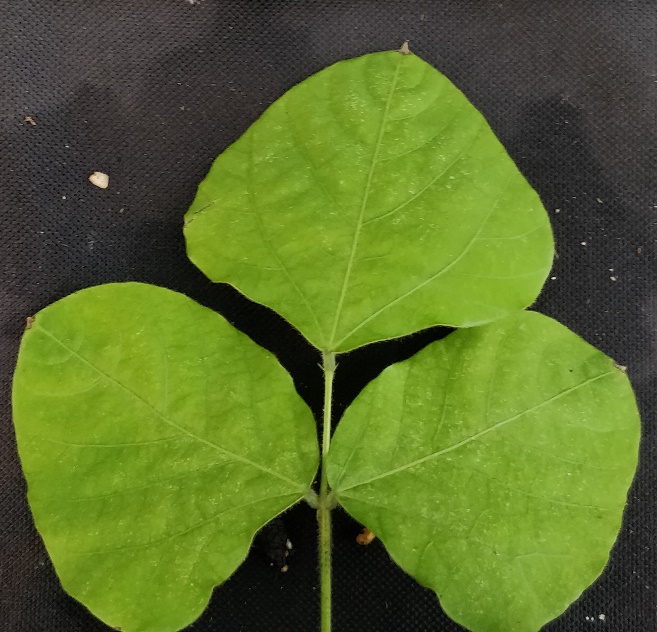

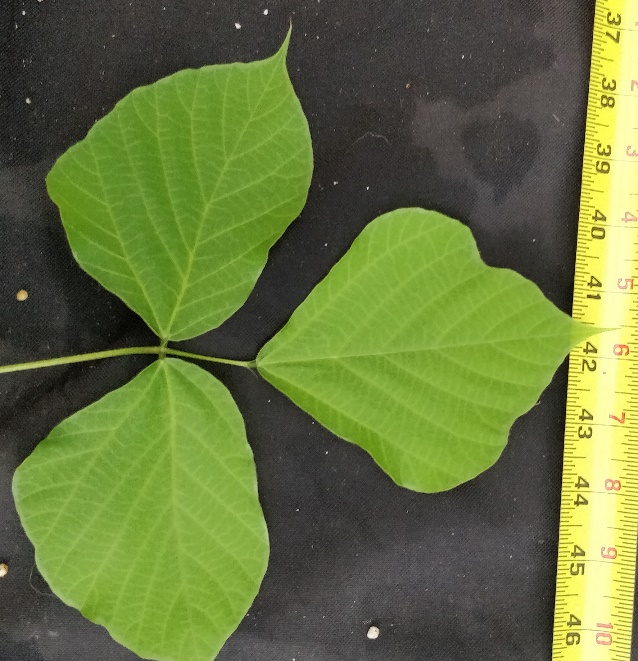


**Supplemental Figure 2.** Leaves from USDA PI 9227 *P. m. lobata* plants

**Supplemental Figure 3.** Percent compositon of six common isoflavones in each of the seven accessions.


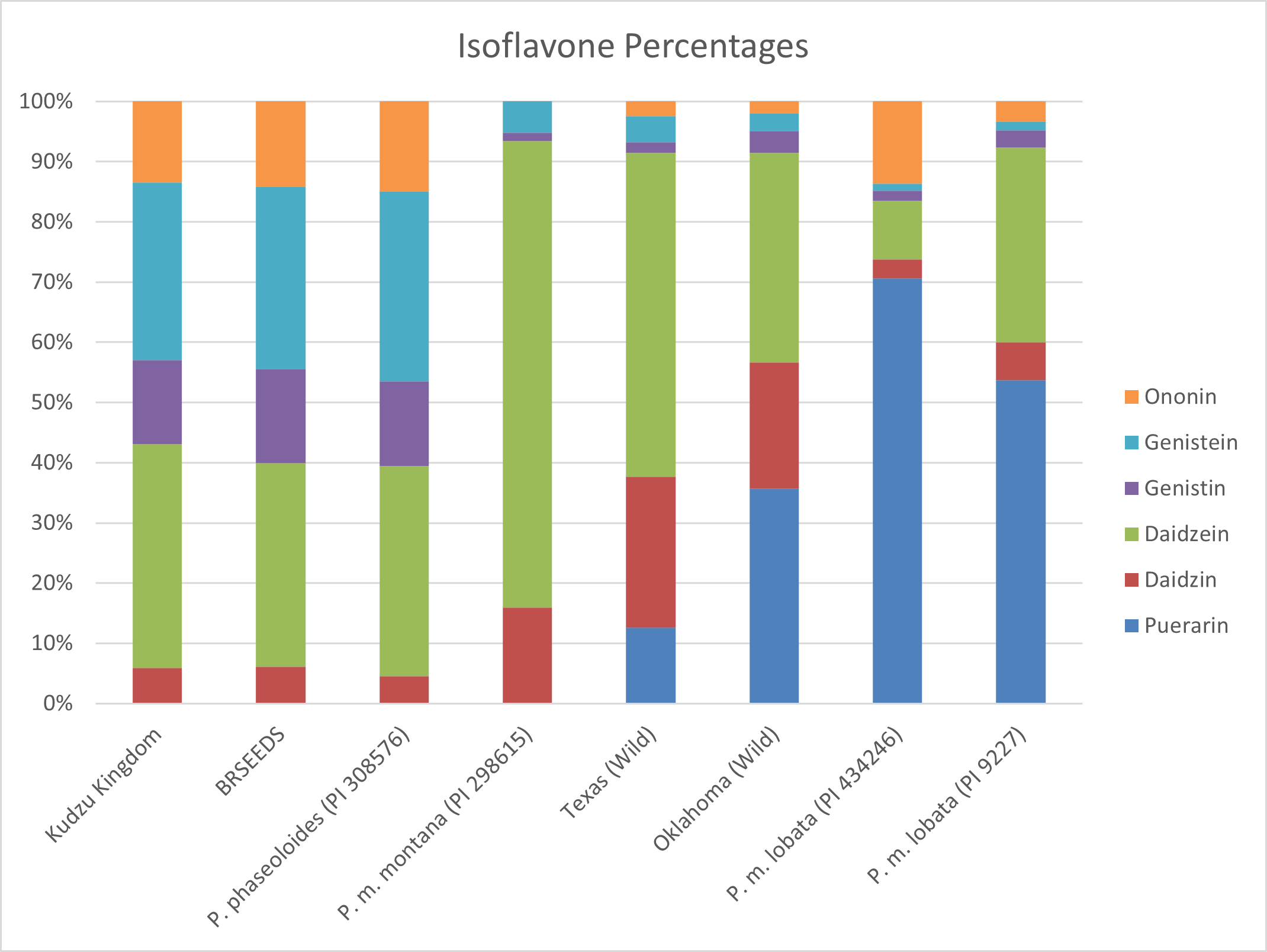


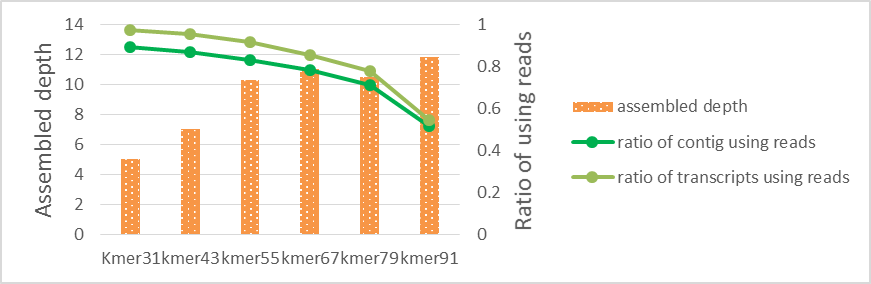

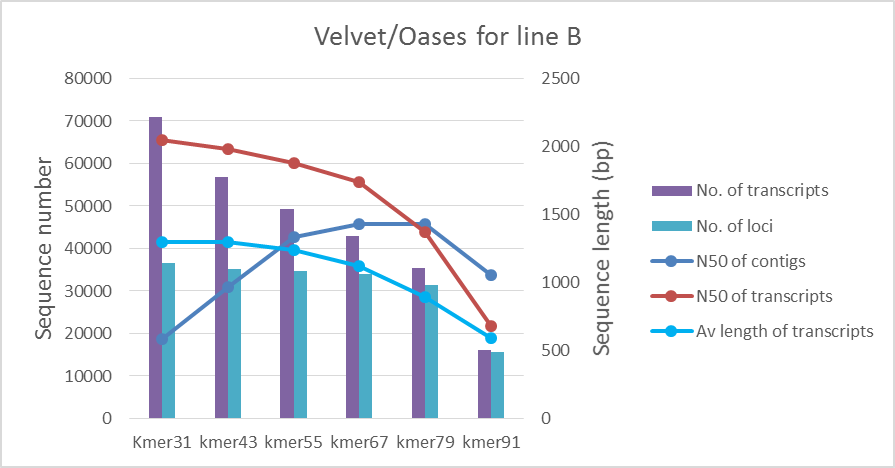

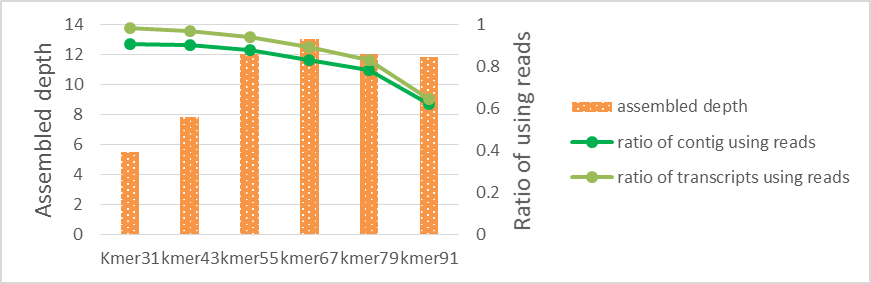


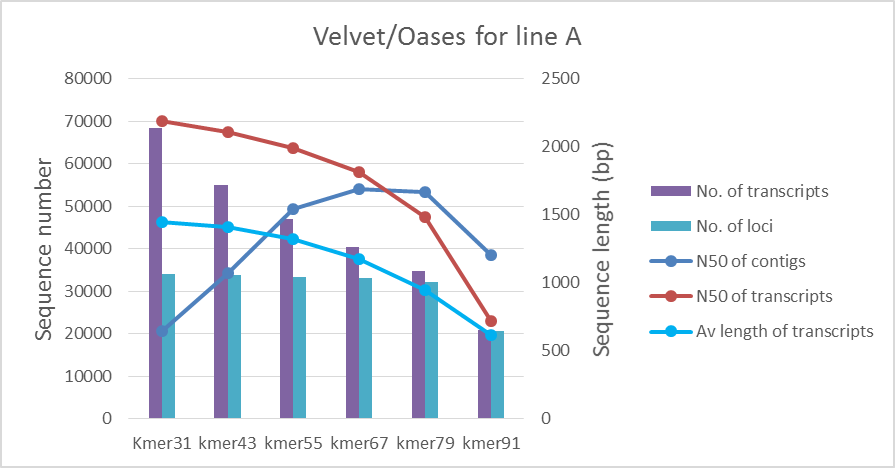


*P. phaseoloides*

*P. m. lobata*

**Supplemental Figure 4.** Quality measurements for Velvet/Oases assemblies


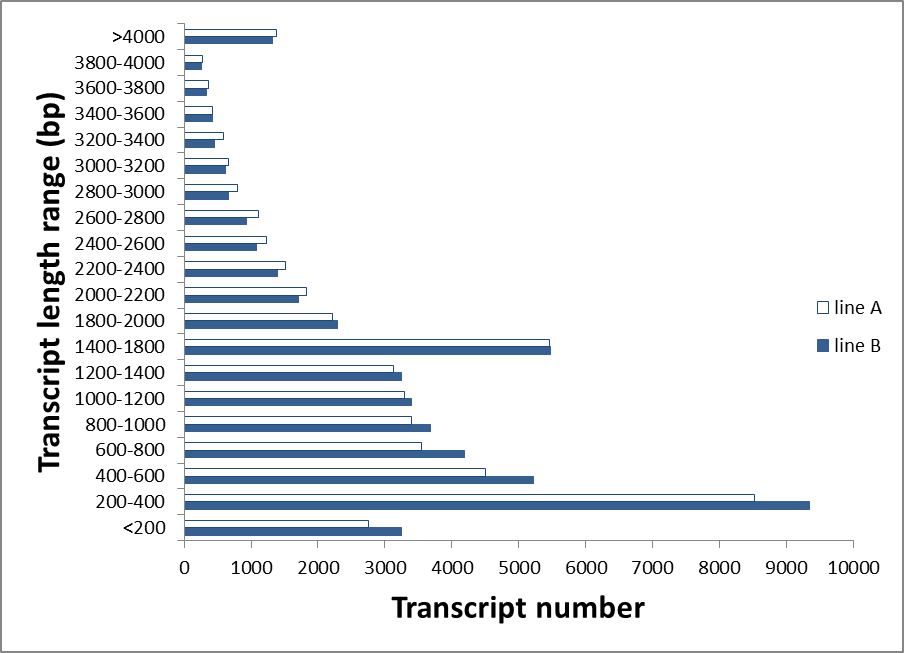


*P. m. lobata*

*P. phaseoloides*


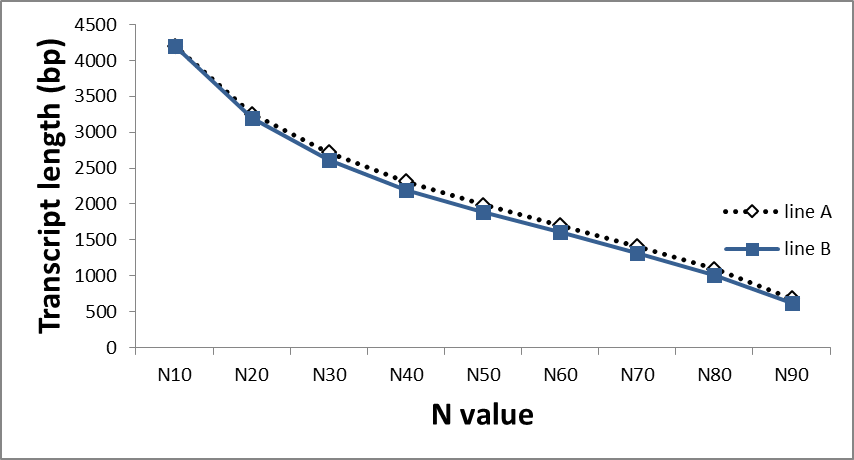


*P. m. lobata*

*P. phaseoloides*

**Supplemental Figure 5.** Length distribution of the assembled transcripts in *P. phaseoloides* and *P. m. lobata*


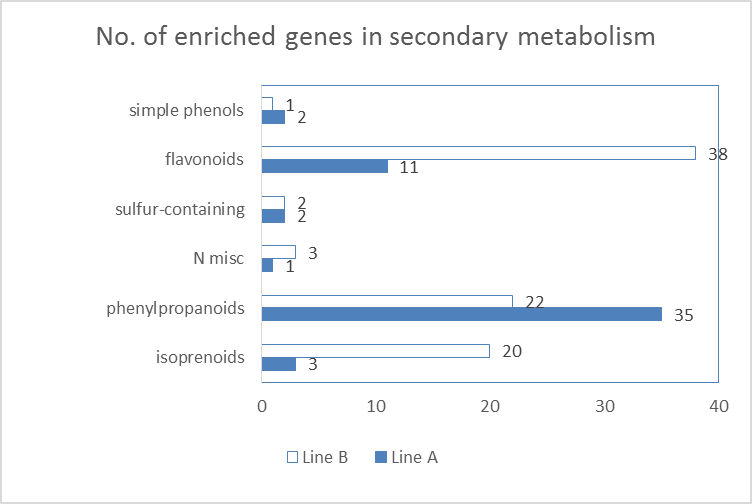

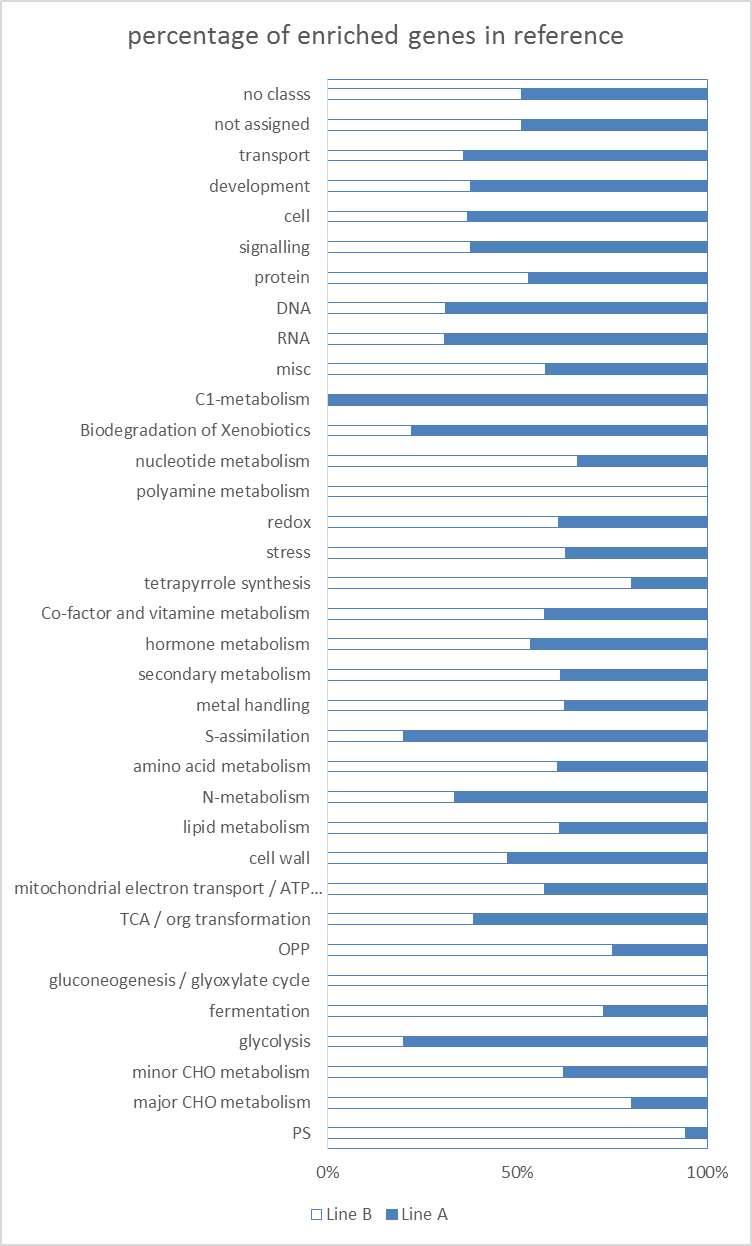

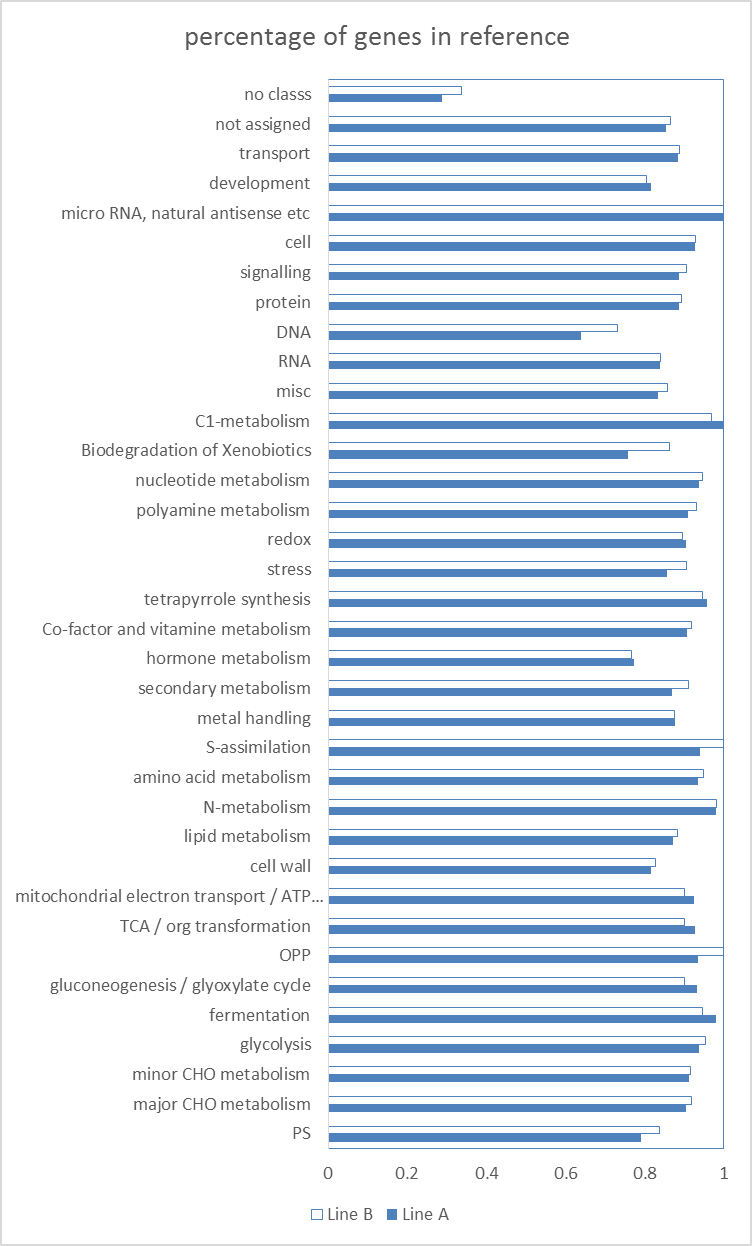

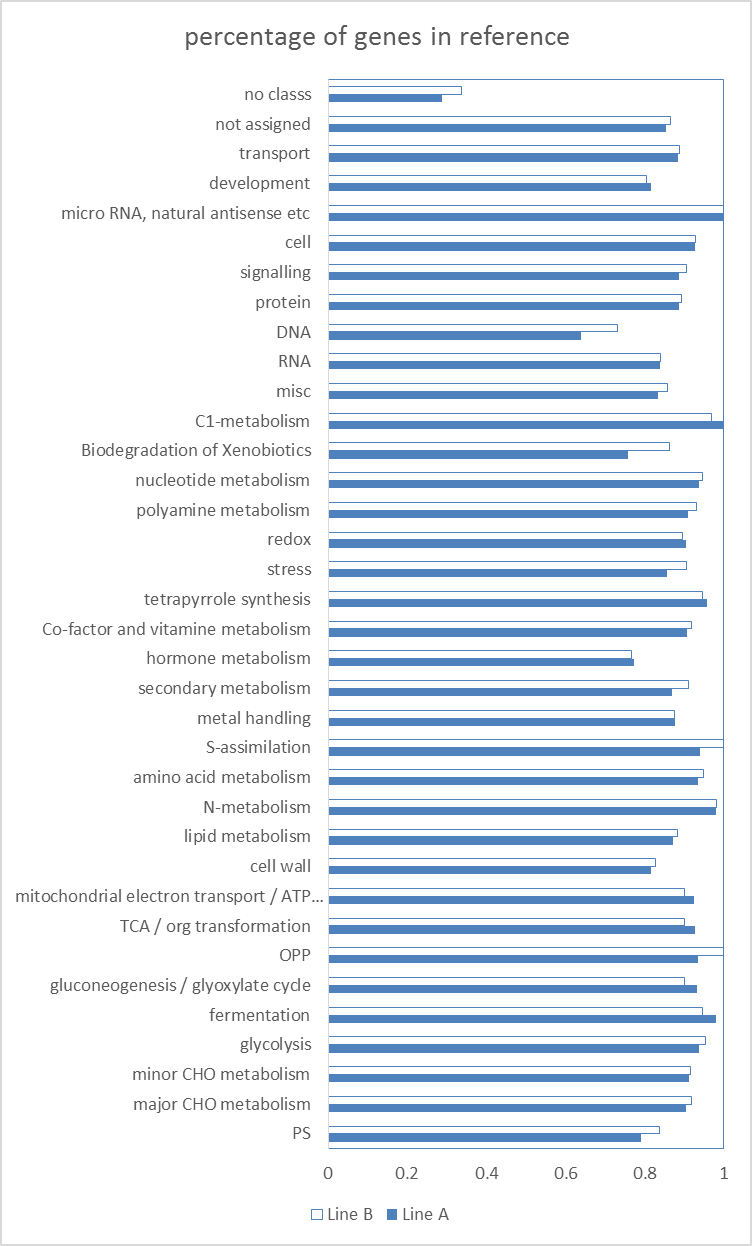


**Supplemental Figure 6.** Pathway representation analysis of the soybean transcripts mapped by *Pueraria* reads. (A) Coverage of the functional classes in soybean transcripts mapped by line A (*P. phaseoloides*) and B (*P.m. lobata*). (B) Coverage of the functional classes in differentially accumulated transcripts between line A (*P. phaseoloides*) and B (*P.m. lobata*). (C) Comparison of differentially-expressed transcripts involved in secondary metabolism between line A (*P. phaseoloides*) and B (*P.m. lobata*).

C

B

A

| Supplemental Table 1. ITS2 nucleotide changes between *P. m. lobata and P. phaseoloides* | |
| --- | --- |
| Nucleotide changes | |
| *P. m. lobata* | *P. phaseoloides* |
| CAACGCA | TCCTCAC |
| ACA | CAT |
| ACT | CATG |
| CG | GT |
| CGTTGCGTG | ATGCAGTAA |
| T | C |
| C | T |
| C | T |
| A | G |
| G | A |
| G | A |
| G | C (S) |
| T | C |
| C | T |
| C (S)AC | TGT |
| A | C |
| C | T |
| CGCTCGAGACC | TTACGCTCGAG |
| ATC | CCG |
| CGCGCT | TCACGA |
| A | T |
| GT | TC |
| C | A |
| GAA | TCC |
| CT | AC |
| TA | CT |
| A | G |
| CGC | TCT |
| C | G |
| T | A |
| TC | AG |
| GG | AA |
| G (K) | A |

| Supplemental Table 2. ITS2 insertions/deletions between *P. m. lobata* and *P. phaseoloides* | |
| --- | --- |
| Indels | |
| *P. m. lobata* | *P. phaseoloides* |
| CCACACGA | -------- |
| GATTGATGAC | ---------- |

| Supplemental Table 3. ITS2 nucleotide changes between *P. m. lobata* and *P. m. montana* | |
| --- | --- |
| Nucleotide changes | |
| *P. m. lobata* | *P. m. montana* |
| CAA | TCC |
| CA | TC |
| CG | AT |
| C | T |
| T | C |
| T | A |
| C | T |
| C | T |
| C | T |
| G | C |
| C | T |
| T | C |
| C | T |
| A | G |
| A | G |
| T | C |
| T | C |
| A | C |
| A | G |
| A | G |
| TG | GA |
| GA | CC |
| TCGGTCCGCGAA | GGACCAGTTCCG |
| GAC | CGA |
| TACA | CTGC |
| GC | CT |
| TC | CG |
| TCT | AAG |
| G (K) | A |

| Supplemental Table 4. ITS2 insertions/deletions between *P. m. lobata* and *P. m. montana* | |
| --- | --- |
| Indels | |
| *P. m. lobata* | *P. m. montana* |
| TCCCACACGACGGCCGTTG | ------------------- |
| G | - |
| CTGATTGATGA | ----------- |

| Supplemental Table 5. ITS2 nucleotide changes between *P. phaseoloides* and *P. m. montana* | |
| --- | --- |
| Nucleotide changes | |
| *P. phaseoloides* | *P. m. montana* |
| TCAC | CGCA |
| CAT | ATC |
| C | A |
| GCG | TGC |
| A | G |
| C | A |
| G | A |
| C | T |
| A | G |
| A | C |
| C (S) | G |
| C | T |
| C | T |
| T | C |
| A | G |
| T | C |
| T | C |
| T | C |
| T | C |
| T | C |
| GA | AG |
| A | G |
| T | C |
| TC | CG |
| T | A |
| C | T |
| CTC | ACT |
| A | C |
| GAC | CGA |
| CG | GC |
| T | C |
| T | C |
| CAG | AGC |
| AA | GG |

| Supplemental Table 6. ITS2 insertions/deletions between *P. phaseoloides* and *P. m. montana* | |
| --- | --- |
| Indels | |
| *P. phaseoloides* | *P. m. montana* |
| TGCATGCAGTA | ----------- |
| TTA | --- |
| -- | AC |
| A | - |

| **Supplemental Table 7.** | | | | | | | | | | | | |
| --- | --- | --- | --- | --- | --- | --- | --- | --- | --- | --- | --- | --- |
|  | **Assembly Statistics (Velvet/Oases) for *P. phaseoloides*** | | | | | | **Assembly Statistics (Velvet/Oases) for *P. m. lobata*** | | | | | |
|  | *Kmer31* | *kmer43* | *kmer55* | *kmer67* | *kmer79* | *kmer91* | *Kmer31* | *kmer43* | *kmer55* | *kmer67* | *kmer79* | *kmer91* |
| *N50 of contigs* | 646 | 1071 | 1540 | 1689 | 1666 | 1206 | 583 | 967 | 1337 | 1432 | 1429 | 1053 |
| *N50 of transcripts* | 2186 | 2109 | 1988 | 1813 | 1479 | 719 | 2050 | 1979 | 1881 | 1737 | 1374 | 679 |
| *Av length of transcripts* | 1443.842 | 1405.693 | 1320.158 | 1173.198 | 947.2778 | 614.0364 | 1299.892 | 1299.446 | 1239.23 | 1119.002 | 894.4756 | 593.5302 |
| *No. of transcripts* | 68332 | 55060 | 47011 | 40441 | 34819 | 20827 | 70987 | 56889 | 49277 | 42910 | 35370 | 16227 |
| *No. of loci* | 33927 | 33731 | 33221 | 33000 | 32190 | 20555 | 36586 | 35120 | 34677 | 33987 | 31501 | 15705 |
| *Assembled depth* | 5.5 | 7.8 | 12 | 13 | 12 | 11.8 | 5 | 7 | 10.3 | 11 | 10.5 | 11.8 |
| *Ratio of contig using reads* | 0.905117 | 0.899327 | 0.877904 | 0.831324 | 0.782235 | 0.622636 | 0.891749 | 0.866954 | 0.82937 | 0.782431 | 0.709322 | 0.517104 |
| *Ratio of transcripts using reads* | 0.98177 | 0.967532 | 0.938289 | 0.892349 | 0.83187 | 0.642554 | 0.974663 | 0.955725 | 0.917794 | 0.855116 | 0.775871 | 0.542875 |

**Supplemental Table 8.** Putative SSRs from transcripts of *P. phaseoloides* and *P. m. lobata* (present in pdf document)

| **Supplemental Table 9.** Statistics of *Pueraria* reads mapped to soybean by BLAST | | | |  |
| --- | --- | --- | --- | --- |
|  | Clean reads | Mapped reads | Map ratio | Number of mapped proteins |
| *P. phaseoloides* | 19007105 | 12369952 | 0.650807 | 44130 |
| *P. m. lobata* | 16445640 | 10797298 | 0.656545 | 45100 |
