# Supplementary material for: Identification of Pueraria spp. through DNA barcoding and comparative transcriptomics
Source: BMC Plant Biol. 2022 Jan 3;22:10. doi: 10.1186/s12870-021-03383-x (PMC8722073; doi:10.1186/s12870-021-03383-x)
Supplement: Supplementary file 2 — Additional file 2: Supplemental Dataset 1. Putative SSRs from transcripts of P. phaseoloides and P. m. lobata. [file 12870_2021_3383_MOESM2_ESM.pdf]

Supplemental Dataset 1: Frequency of putative SSR repeat types (considering sequence complementarity) from transcripts of *P. phaseoloides* and *P. m. lobata*

Pueraria phaseoloides

**Repeats**	**5**	**6**	**7**	**8**	**9**	**10**	**>=11**	**total**
A/T	-	-	-	-	-	1314	1891	3205
C/G	-	-	-	-	-	11	30	41
AC/GT	-	-	105	68	41	20	37	271
AG/CT	-	-	337	268	256	159	653	1673
AT/AT	-	-	62	49	23	19	44	197
CG/CG	-	-	2				0	2
AAC/GTT	181	101	38	39	17	13	15	404
AAG/CTT	348	182	138	77	57	35	65	902
AAT/ATT	120	60	33	19	8	10	15	265
ACC/GGT	131	89	40	17	11	3	4	295
ACG/CGT	55	20	8	9	1	4	1	98
ACT/AGT	27	6	4				0	37
AGC/CTG	134	60	34	24	12	7	9	280
AGG/CCT	167	92	51	35	14	9	9	377
ATC/ATG	160	85	40	16	11	6	10	328
CCG/CGG	133	69	33	19	11	4	0	269
AAAC/GTTT	7	3	1		1		0	12
AAAG/CTTT	30	14	1	5		1	0	51
AAAT/ATTT	24	6	2	1			0	33
AACC/GGTT	1	1	1				0	3
AACG/CGTT	1						0	1
AACT/AGTT	2	2					0	4
AAGC/CTTG	-	2					0	2
AAGG/CCTT	6		1				0	7
AATC/ATTG	3						0	3
AATG/ATTC	16	4	2				0	22
AATT/AATT	5	5	1			1	0	12
ACAT/ATGT	3			1			0	4
ACTC/AGTG	11	9	2	2	1		0	25
AGAT/ATCT	6	3	3	1			0	13
AGCC/CTGG	5						0	5
AGGG/CCCT	2	2					0	4
ATCC/ATGG	-	1		1			0	2
ATGC/ATGC	-		1				0	1
AAAAC/GTTTT	4						0	4
AAAAG/CTTTT	5	3	1				0	9
AAAAT/ATTTT	7	1					0	8
AAACC/GGTTT	3	1					0	4
AAACG/CGTTT	1						0	1

AAAGC/CTTTG	-	1					0	1
AAAGG/CCTTT	2						0	2
AAATC/ATTTG	2						0	2
AAATG/ATTTC	2						0	2
AAATT/AATTT	1						0	1
AACAC/GTGTT	17	2	3		1		0	23
AACAG/CTGTT	5	2					0	7
AACAT/ATGTT	2	1					0	3
AACCC/GGGTT	4						0	4
AACCG/CGGTT	-	1					0	1
AACGC/CGTTG	1						0	1
AACTC/AGTTG	1						0	1
AAGAC/CTTGT	1						0	1
AAGAG/CTCTT	12	5		1			0	18
AAGGG/CCCTT	3						0	3
AAGGT/ACCTT	1						0	1
AAGTG/ACTTC	3						0	3
AATAG/ATTCT	3						0	3
AATCC/ATTGG	1						0	1
AATCG/ATTCG	1						0	1
AATTC/AATTG	3						0	3
ACACC/GGTGT	1	3					0	4
ACACT/AGTGT	2						0	2
ACAGC/CTGTG	5						0	5
ACATG/ATGTC	1						0	1
ACCAT/ATGGT	1						0	1
ACCCC/GGGGT	1						0	1
ACCGC/CGGTG	1						0	1
ACCTC/AGGTG	1						0	1
ACGAG/CGTCT	-			1			0	1
ACTCC/AGTGG	-	1					0	1
AGAGG/CCTCT	1	1					0	2
AGATG/ATCTC	2	1					0	3
AGCAT/ATGCT	-	1					0	1
AGCCC/CTGGG	1						0	1
AGCTC/AGCTG	1						0	1
ATATC/ATATG	1						0	1
ATCCC/ATGGG	1						0	1
ATCGC/ATGCG	1						0	1
CCCGG/CCGGG	1						0	1
AAAAAG/CTTTTT	1		1				0	2
AAAAAT/ATTTTT	-		1				0	1
AAAACC/GGTTTT	3						0	3

AAAAGG/CCTTTT	3						0	3
AAAATG/ATTTTC	1	1					0	2
AAACAC/GTGTTT	1						0	1
AAACCC/GGGTTT	5		1				0	6
AAACGC/CGTTTG	1	1					0	2
AAACTC/AGTTTG	1						0	1
AAAGAG/CTCTTT	5	1					0	6
AAAGAT/ATCTTT	1						0	1
AAAGCC/CTTTGG	1	1					0	2
AAAGGC/CCTTTG	1						0	1
AAAGGG/CCCTTT	1						0	1
AAAGGT/ACCTTT	1						0	1
AAATCC/ATTTGG	2	1					0	3
AAATCG/ATTTCG	1	1					0	2
AAATGG/ATTTCC	1						0	1
AAATTG/AATTTC	1		1				0	2
AAATTT/AAATTT	1						0	1
AACACC/GGTGTT	5						0	5
AACACG/CGTGTT	1		1				0	2
AACAGC/CTGTTG	1	1					0	2
AACATG/ATGTTC	1						0	1
AACCAG/CTGGTT	-		1				0	1
AACCAT/ATGGTT	2						0	2
AACCCC/GGGGTT	2						0	2
AACCCG/CGGGTT	4				1		0	5
AACCCT/AGGGTT	6		2	1			0	9
AACCTC/AGGTTG	1						0	1
AACCTG/AGGTTC	1						0	1
AACCTT/AAGGTT	-	1					0	1
AACGCC/CGTTGG	2						0	2
AACGTC/ACGTTG	1						0	1
AACTCC/AGTTGG	2	1	2				0	5
AACTCG/AGTTCG	1						0	1
AACTGC/AGTTGC	1						0	1
AACTTC/AAGTTG	3						0	3
AACTTG/AAGTTC	1						0	1
AAGAGG/CCTCTT	6	1	2				0	9
AAGATG/ATCTTC	2		1				0	3
AAGCAC/CTTGTG	-		1				0	1
AAGCAG/CTGCTT	8						0	8
AAGCCC/CTTGGG	3						0	3
AAGCCG/CGGCTT	1	1	1				0	3
AAGGAG/CCTTCT	3	2		1			0	6

AAGGGG/CCCCTT	1	1					0	2
AAGGTG/ACCTTC	1						0	1
AAGTAG/ACTTCT	1						0	1
AAGTGC/ACTTGC	1						0	1
AAGTGG/ACTTCC	1						0	1
AATAGC/ATTGCT	1		1				0	2
AATCAG/ATTCTG	-	1					0	1
AATCCC/ATTGGG	2	1					0	3
AATCCG/ATTCGG	-	1					0	1
AATCGC/ATTGCG	1						0	1
AATCGG/ATTCCG	1						0	1
AATCTC/AGATTG	-		1				0	1
AATGGG/ATTCCC	1						0	1
AATGTG/ACATTC	1						0	1
AATTCC/AATTGG	2						0	2
ACAGCC/CTGTGG	1	1					0	2
ACAGCT/AGCTGT	1						0	1
ACAGTC/ACTGTG	1						0	1
ACATGG/ATGTCC	-	1	1				0	2
ACCACG/CGTGGT	1						0	1
ACCAGC/CTGGTG	2	1		1			0	4
ACCATC/ATGGTG	2		1				0	3
ACCATG/ATGGTC	1		2				0	3
ACCCGG/CCGGGT	1						0	1
ACCCTC/AGGGTG	3						0	3
ACCGCC/CGGTGG	3						0	3
ACCGGC/CCGGTG	1						0	1
ACCGTC/ACGGTG	2	1	1				0	4
ACCTCC/AGGTGG	1						0	1
ACCTCG/AGGTCG	-	1					0	1
ACCTGC/AGGTGC	4						0	4
ACCTGG/AGGTCC	1						0	1
ACGAGG/CCTCGT	1						0	1
ACGATC/ATCGTG	1						0	1
ACGCCG/CGGCGT	1	1					0	2
ACGGAG/CCGTCT	1						0	1
ACGGCG/CCGTCG	-	2				1	0	3
ACGGCT/AGCCGT	-	1					0	1
ACGGGC/CCCGTG	-	1					0	1
ACTAGC/AGTGCT	1						0	1
ACTCAT/AGTATG	-	1					0	1
ACTCCC/AGTGGG	1						0	1
ACTCTC/AGAGTG	1						0	1

ACTGAG/AGTCTC	1						0	1
ACTGCC/AGTGGC	1						0	1
ACTGCG/AGTCGC	1						0	1
ACTGGC/AGTGCC	1						0	1
ACTGGG/AGTCCC	1						0	1
AGAGCC/CTCTGG	1						0	1
AGAGGC/CCTCTG	2						0	2
AGAGGG/CCCTCT	2	1					0	3
AGATCC/ATCTGG	1						0	1
AGCAGG/CCTGCT	1						0	1
AGCATC/ATGCTG	2	1					0	3
AGCATG/ATGCTC	-	1					0	1
AGCCCC/CTGGGG	1			1			0	2
AGCCCG/CGGGCT	4						0	4
AGCCGG/CCGGCT	1	1		1			0	3
AGCCTC/AGGCTG	2	1		1			0	4
AGCCTG/AGGCTC	2						0	2
AGGATG/ATCCTC	1						0	1
AGGCCC/CCTGGG	2	1					0	3
AGGCGG/CCGCCT	3						0	3
AGGGGC/CCCCTG	2						0	2
ATCCGG/ATCCGG	1						0	1

Pueraria montana lobata

Repeats	**5**	**6**	**7**	**8**	**9**	**10**	**>=11**	**total**
A/T	-	-	-	-	-	972	1638	2610
C/G	-	-	-	-	-	10	5	15
AC/GT	-	-	74	33	32	5	20	164
AG/CT	-	-	271	201	147	101	142	862
AT/AT	-	-	41	29	16	12	13	111
CG/CG	-	-		1			0	1
AAC/GTT	184	118	53	24	10	2	1	392
AAG/CTT	335	147	85	37	27	14	16	661
AAT/ATT	101	62	17	11	12	3	0	206
ACC/GGT	158	88	50	15	4	3	0	318
ACG/CGT	47	29	9	3		1	1	90
ACT/AGT	29	15	2		1		0	47
AGC/CTG	119	45	20	9	2		1	196
AGG/CCT	125	60	21	11	3	1	0	221
ATC/ATG	167	77	23	16	7	2	2	294
CCG/CGG	106	44	17	6	8		0	181
AAAC/GTTT	7	2		1			0	10

AAAG/CTTT	21	5	7	1	0	34
AAAT/ATTT	7	1			0	8
AACC/GGTT	3	1			0	4
AACG/CGTT	2				0	2
AACT/AGTT	4	1			0	5
AAGG/CCTT	2	2	1		0	5
AAGT/ACTT	1				0	1
AATC/ATTG	4				0	4
AATG/ATTC	2				0	2
AATT/AATT	4				0	4
ACAG/CTGT	2				0	2
ACCG/CGGT	-	1			0	1
ACGC/CGTG	1				0	1
ACTC/AGTG	7	3	1		0	11
ACTG/AGTC	1				0	1
AGAT/ATCT	6	2			0	8
AGCC/CTGG	3				0	3
AGCG/CGCT	1				0	1
AGCT/AGCT	1				0	1
AGGC/CCTG	1				0	1
AGGG/CCCT	5				0	5
ATCC/ATGG	2				0	2
AAAAC/GTTTT	2				0	2
AAAAG/CTTTT	3				0	3
AAAAT/ATTTT	1	1			0	2
AAACC/GGTTT	1				0	1
AAACG/CGTTT	-	1			0	1
AAACT/AGTTT	1				0	1
AAAGC/CTTTG	1				0	1
AAATC/ATTTG	1	1			0	2
AACAC/GTGTT	9	2			0	11
AACAG/CTGTT	1	1	1		0	3
AACCC/GGGTT	1	1			0	2
AACTC/AGTTG	2		1		0	3
AACTG/AGTTC	1				0	1
AAGAG/CTCTT	7	7			0	14
AAGGC/CCTTG	2				0	2
AAGGG/CCCTT	-	2			0	2
AATAC/ATTGT	1				0	1
AATAT/ATATT	1				0	1
AATCC/ATTGG	1				0	1
AATCG/ATTCG	1				0	1
AATTC/AATTG	2				0	2

ACACC/GGTGT	5			0	5
ACACG/CGTGT	-		1	0	1
ACACT/AGTGT	1			0	1
ACCCG/CGGGT	1			0	1
ACCTC/AGGTG	1			0	1
ACGAG/CGTCT	1			0	1
ACGGC/CCGTG	1			0	1
ACTGC/AGTGC	1	1		0	2
AGAGC/CTCTG	1	1		0	2
AGAGG/CCTCT	2			0	2
AGATC/ATCTG	1			0	1
AGATG/ATCTC	1			0	1
ATCCC/ATGGG	1			0	1
ATCCG/ATCGG	1			0	1
ATCGC/ATGCG	2			0	2
AAAACC/GGTTTT	3			0	3
AAACAC/GTGTTT	1			0	1
AAACCC/GGGTTT	3			0	3
AAACTG/AGTTTC	1			0	1
AAAGCC/CTTTGG	2			0	2
AAAGGG/CCCTTT	1			0	1
AAAGTG/ACTTTC	1			0	1
AAATAT/ATATTT	1			0	1
AAATCC/ATTTGG	1			0	1
AAATCG/ATTTCG	1			0	1
AAATGC/ATTTGC	-	1		0	1
AACACC/GGTGTT	1	2		0	3
AACCAG/CTGGTT	3	1		0	4
AACCCG/CGGGTT	1	2		0	3
AACCCT/AGGGTT	3	1		0	4
AACCTC/AGGTTG	1	1		0	2
AACCTG/AGGTTC	-	1		0	1
AACGCC/CGTTGG	1	1		0	2
AACGGG/CCCGTT	1			0	1
AACTGC/AGTTGC	1			0	1
AACTGG/AGTTCC	4			0	4
AAGACG/CGTCTT	1			0	1
AAGAGG/CCTCTT	3			0	3
AAGATG/ATCTTC	2			0	2
AAGCAG/CTGCTT	2			0	2
AAGCCC/CTTGGG	-	1		0	1
AAGGAG/CCTTCT	-	1		0	1
AAGTCC/ACTTGG	1			0	1

AAGTGC/ACTTGC	1						0	1
AAGTGT/ACACTT	-	1					0	1
AATACC/ATTGGT	-	1					0	1
AATCGG/ATTCCG	1						0	1
AATGTG/ACATTC	1						0	1
AATTAC/AATTGT	1						0	1
AATTCC/AATTGG	1						0	1
ACAGCC/CTGTGG	-	1					0	1
ACAGGC/CCTGTG	1						0	1
ACAGTC/ACTGTG	1						0	1
ACATGG/ATGTCC	2						0	2
ACCACG/CGTGGT	1						0	1
ACCATC/ATGGTG	1						0	1
ACCATG/ATGGTC	1						0	1
ACCCTC/AGGGTG	1	1					0	2
ACCCTG/AGGGTC	1						0	1
ACCGAG/CGGTCT	1						0	1
ACCGCC/CGGTGG	1		1				0	2
ACCGTC/ACGGTG	1						0	1
ACCGTG/ACGGTC	1						0	1
ACCTCC/AGGTGG	-	2					0	2
ACCTGC/AGGTGC	2						0	2
ACGAGC/CGTGCT	1						0	1
ACTAGC/AGTGCT	1						0	1
ACTGCC/AGTGGC	1						0	1
AGAGCC/CTCTGG	2						0	2
AGATCG/ATCTCG	1						0	1
AGATGG/ATCTCC	-	1					0	1
AGCAGG/CCTGCT	-	1					0	1
AGCATC/ATGCTG	1						0	1
AGCATG/ATGCTC	-	1					0	1
AGCCCG/CGGGCT	-			1			0	1
AGCCTC/AGGCTG	1						0	1
AGCCTG/AGGCTC	3	1					0	4
AGCGGC/CCGCTG	1						0	1
AGCTCC/AGCTGG	1						0	1
AGGCAT/ATGCCT	1						0	1
AGGCCC/CCTGGG	1						0	1
